# A periodic dodecagonal supertiling by self-assembly of star-shaped molecules in the liquid crystalline state

**DOI:** 10.1038/s42004-020-0314-1

**Published:** 2020-06-01

**Authors:** Marco Poppe, Changlong Chen, Silvio Poppe, Feng Liu, Carsten Tschierske

**Affiliations:** 1 Institute of Chemistry, Martin-Luther-University Halle-Wittenberg, Kurt-Mothes-Straße 2, 06120 Halle, Germany; 2 State Key Laboratory for Mechanical Behaviour of Materials, Shaanxi International Research Center for Soft Matter, Xi’an Jiaotong University, 710049 Xi’an, People’s Republic of China

**Keywords:** Liquid crystals, Self-assembly, Molecular self-assembly, Organic molecules in materials science

## Abstract

Molecular tessellations are known in solid state systems and their formation is often induced or supported by a periodic surface lattice. Here we discover a complex tessellation on the 10 nm length scale, spontaneously formed in the highly dynamic liquid crystalline state. It is composed of overlapping dodecagonal supertiles combining prismatic cells with triangular and square cross sections. This complex honeycomb occurs between a triangular honeycomb at high and a square at low temperature, being opposite to the sequence expected for a thermal expansion of the side chains in the prismatic cells. Formation of the supertiles is supported by the segregation of alkyl chains with different length. The emergent behaviour of this complex soft matter structure is demonstrated, and intriguing connections between self-assembly on surfaces, in liquid crystals, and in block copolymers are drawn. Moreover, the tessellation represents a close approximant of the elusive columnar liquid quasicrystal with dodecagonal symmetry.

## Abstract (web summary)

Bolaamphiphiles can form liquid crystalline phases with unusual honeycomb-like structures. Here a periodic dodecagonal supertiled structure is observed in liquid crystals, emerging at the transition from triangular to square tiling patterns.

## Introduction

**Fig. 1:**
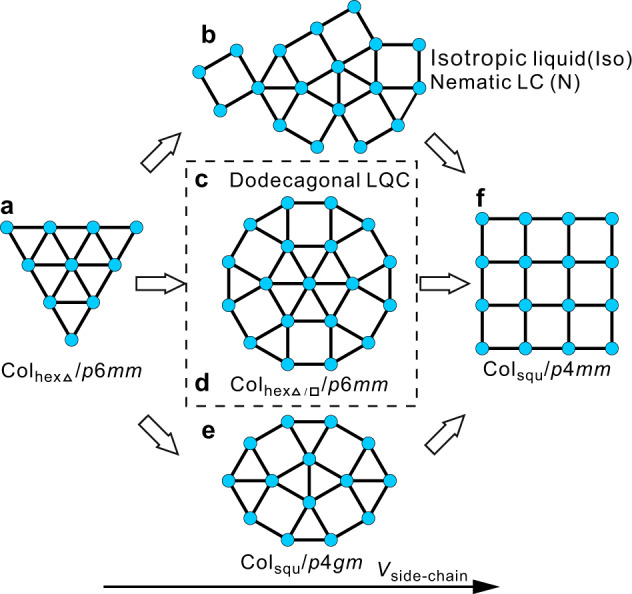
Distinct modes of transitions from triangular to square honeycombs. Transition from triangular (**a**) to square (**f**) honeycomb LC phases occurring via aperiodic (**b**), quasiperiodic (**c**) or long range periodic (**d**, **e**) intermediate structures^4,11,12^. The shown 2D tiling patterns represent cuts through the 3D honeycomb phases perpendicular to the column long axis (*c* axis).

The generation of complex superstructures by self-assembly of molecular building blocks is a powerful tool for preparation of new materials with advanced properties and functions, as well as it is of importance for the understanding of the fundamentals of development of structural complexity in biosystems^1,2^. Liquid crystalline (LC) phases with unprecedented complexity have recently been achieved by the family of T-/X-shaped polyphilic molecules composed of a rod-like *π*-conjugated core, two terminally attached hydrogen bonding glycerol groups and one or two lipophilic lateral chains, among them fluids composed of polygonal honeycombs^3–9^. In these LC honeycombs (Fig. 1), rod-like molecules form walls, fused together at the ends by the hydrogen bonding networks of the terminal glycerols (blue dots) and the resulting prismatic cells are filled by the lateral chains. Emergence of new tiling patterns based on geometrical frustration can be expected at the cross-over between triangular and square honeycombs (Fig. 1a, f)^10,11^. In previous attempts, either the formation of less ordered cybotactic isotropic or nematic phases (Fig. 1b) or a columnar LC with *p*4*gm* lattice, combining triangular and square tiles in a ratio 2:1 (Fig. 1e), were observed^11,12^.

Herein we report the formation of a new LC honeycomb involving dodecagonal supertiles and representing a close approximant of the elusive columnar liquid quasicrystal (LQC) with dodecagonal symmetry (Fig. 1c, d)^13^. It is shown that this complex honeycomb is induced by the combination of steric and geometric frustration, and can transform either to a triangular honeycomb on heating or to a square one on cooling. Whereas the excess space available for the side chains in the square honeycombs is reduced by tilting of the rods forming the honeycomb walls, there is an overcrowding by those chains in the triangular cells, which therefore become defective, locally fusing to larger rhombic cells. The importance of the entropic stabilization by those rhombs, and of the segregation of the long from the short alkyl chains, for the development of the observed structural complexity are discussed in detail.

## Results

### Molecular design

**Fig. 2:**
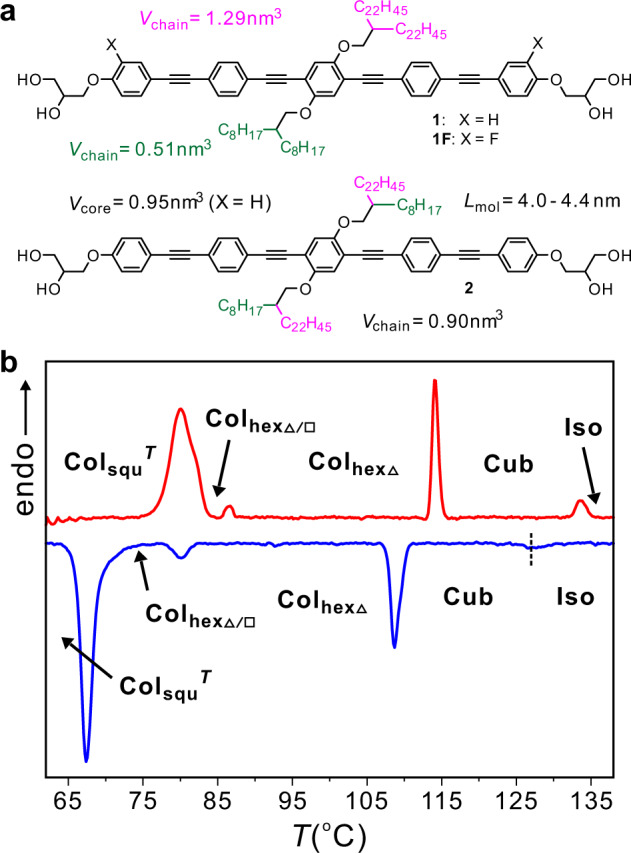
Molecular structures of the investigated compounds **1**, **1F** and **2** and DSC trace of compound 1. **a** Molecular structures with molecular parameters. **b** DSC traces of the second heating and cooling runs (10 K min^−1^) of compound **1**, measured between 60 and 140 °C to avoid crystallization; for the complete DSCs over a broader temperature range extending to lower temperatures, see Supplementary Fig. 1a.

To this end, three compounds were precisely designed based on a bolaamphiphilic oligo(phenylene ethynylene) (OPE) platform^14^ with a defined length of *L*_mol_ ≈ 4.2 ± 0.2 nm and having sticky ends ensured by hydrogen bonding between glycerol groups. A soft periphery is provided by attachment of two branched alkyl chains positioned at opposite sides of the OPE rods (Fig. 2a). The total volume of the alkyl side chains was particularly chosen to be too large for the triangular, but also too small for the square cells formed by the OPE cores. The two lateral chains either have very different length and volume (compounds **1** and **1F**) or they are identical, but combine branches with very different length (compound **2**). The synthesis of compound **1** is shown in the Methods section and experimental detail as well as analytical data of all three compounds can be found in the Supplementary Methods. Investigation of the compounds **1**, **1F** and **2** was performed by differential scanning calorimetry (DSC), polarizing optical microscopy (POM) and small/wide-angle X-ray scattering (SAXS and WAXS) as also described in the Methods section.

### Liquid crystalline self-assembly of compound 1

**Fig. 3:**
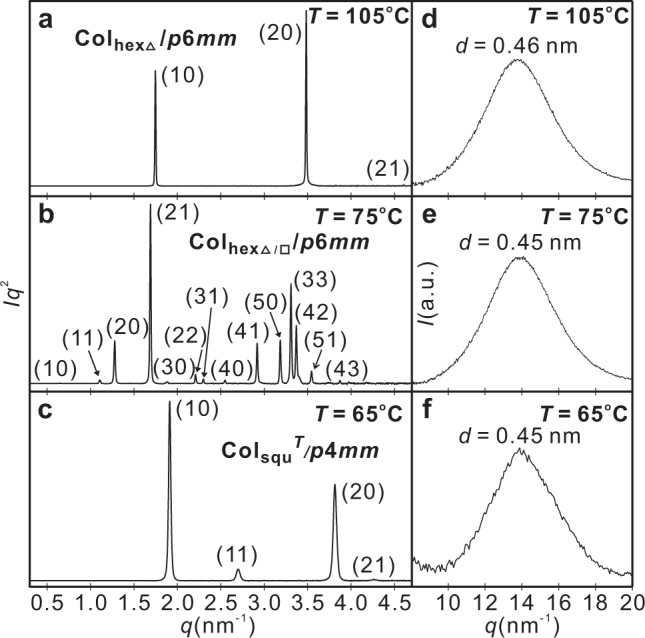
X-ray diffraction patterns of the three LC phases of compound 1. **a**–**c** SAXS diffractograms and **d**–**f** WAXS patterns recorded at the given temperatures (for numerical data, see Supplementary Tables 1–3).

**Fig. 4:**
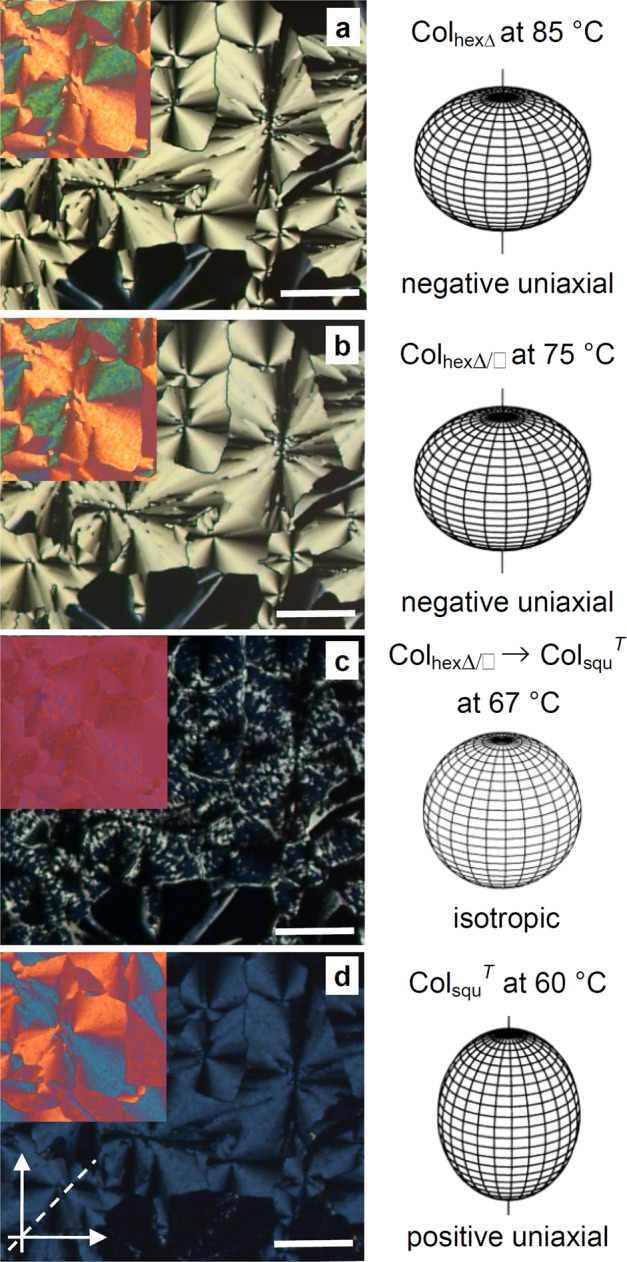
The optical textures of compound 1 as observed between crossed polarizers on cooling from Col_hexΔ_ via Col_hexΔ/□_ phases to Col_squ_^*T*^. **a**–**d** The birefringent planar textures (columns parallel to the surface) and areas with an alignment of the columns perpendicular to the substrate surfaces (optically isotropic dark areas at the bottom). The optical indicatrices of the distinct phases are shown on the right. The scale bars are 100 μm. The insets show the planar textures with additional *λ*-retarder plate. In **d** the orientation of the polarizers (arrows) and the slow indicatrix axis orientation (dotted line) are shown, which are identical for **a**–**d**. In the Col_hexΔ_ and Col_hexΔ/□_ phases (**a**, **b**) the birefringence is negative (the blue shifted fans with λ-retarder plate are parallel to the indicatrix direction), i.e. the main intramolecular *π*-conjugation pathway is on average perpendicular to the column long axis with no visible change in birefringence at the phase transition Col_hexΔ_ − Col_hexΔ/□_ (**a** → **b**). At the first order phase transition to Col_squ_^*T*^ the birefringence suddenly becomes Δ*n* = 0 (**c** and inset) and then weakly positive (**d** the yellow shifted fans are now parallel to the indicatrix direction, see inset in **d**). This indicates that the *π*-conjugation pathway crosses a certain angle at which it changes from being perpendicular to the column direction to predominately parallel. On further cooling the birefringence in the Col_squ_^*T*^ phase remains smaller than in the Col_hex_ phases, indicating that the *π*-conjugated rods retain a tilt by a certain angle with respect to the column long axis. The bright spots and lines in **c** are residues of the coexisting Col_hexΔ/□_ phase at this strongly first order phase transition.

Upon cooling, the isotropic liquid of compound **1** to 132 °C four phase transitions were observed before crystallization at 52 °C (Table 1, Fig. 2b and Supplementary Fig. 1a). In this temperature range, the WAXS patterns show only one diffuse scattering (Fig. 3d–f and Supplementary Fig. 3) as typical for LC phases lacking fixed positions of individual molecules. A significant increase of the viscosity takes place at the first transition at 132 °C, though the sample remains dark between crossed polarizers, i.e. optically isotropic, indicating a transition to a cubic phase (Cub) at this temperature. At the next transition at *T* = 111 °C, a birefringent spherulitic texture develops, being reminiscent for columnar LC phases with the columns aligned parallel to the substrate surfaces (Fig. 4a). The birefringent texture is retained at the phase transition at 78 °C (Fig. 4b), becomes almost optically isotropic at the next transition at 67 °C (Fig. 4c), and then the texture reappears with a very low birefringent bluish color (Fig. 4d). Investigation with an additional lambda-retarder plate shows an inversion of the direction of blue and yellow shifted brushes at the transition at 67 °C (insets in Fig. 4b–d), indicating an inversion of the sign of birefringence from negative above 67 °C to positive below. The dark regions (bottom parts of Fig. 4a–d), where the columns are aligned perpendicular to the substrate surfaces, do not change, indicating that all birefringent LC phases are optically uniaxial, meaning that they represent either hexagonal or square columnar phases. Focus of this work is exclusively on the three birefringent LC phases, while the isotropic cubic phases of compounds **1** and **2**, occurring at the highest temperature will not be considered here.

**Table 1:** LC phases and structural parameters of compounds **1**–**2**.

Comp.	Phase transitions	Phase	*a*/nm	*n*_cell_
**1**	*H*: Cr 98 [50.7] Col_hexΔ_115 [3.7] Cub 134 [0.5] Iso *C*: Iso 132 [–] Cub 111 [-4.6] Col_hexΔ_ 78 [-0.8] Col_hexΔ/□_ 67 [-10.7] Col_squ_^*T*^ 52 [-7.4] Cr	Col_hexΔ_ Col_hexΔ/□_ Col_squ_^*T*^	4.15 11.36 3.29	2.50 20.5 2.65
**1F**	*H*: Cr 83 [68.1] Col_hexΔ/□_ 90 [0.9] Col_hexΔ_ 121 [2.9] Iso *C*: Iso 118 [-2.9] Col_hexΔ_ 81 [-1.6] Col_hexΔ/□_ 39 [-2.4] Cr_1_ 28 [-11.8] Cr_2_	Col_hexΔ_ Col_hexΔ/□_	4.16 11.54	2.40 20.8
**2**	*H*: Cr 118 [113.5] Cub 138 [0.7] Iso *C*: Iso 135 [-0.4] Cub 108 [-4.2] Col_hexΔ_ 76 [-11.4] Cr	Col_hexΔ_	4.17	2.53

Peak temperatures from the first DCS heating (*H*) and cooling (*C*) cycles at 10 K min^−1^ (DSCs of the second scans, see Supplementary Fig. 1), enthalpy values (Δ*H*/kJ mol^−1^) are given in square brackets after the phase-transition temperatures.

*a* Lattice parameter, *n*_*cell*_ number of molecules per unit cell (for calculations and further details, see Supplementary Tables 7–11 in the Supplementary Methods 4.3); *Cr* cystalline solid, Iso isotropic liquid, *Cub* cubic LC phase; *Col*_*hexΔ*_ triangular honeycomb LC phase; *Col*_*hexΔ/□*_ Col_hex_ phase with hexagonal superlattice formed by dodecagonal supertiles (the subscripts Δ and Δ/□ indicate the shape of the prismatic cells forming the tiling pattern of the distinct Col_hex_ phases); *Col*_*squ*_^*T*^ square honeycomb with tilted molecules.

All the birefringent LC phases were thoroughly investigated by in-situ synchrotron SAXS (Methods). The scattering pattern between 111 and 78 °C shows three sharp reflections with a ratio of 1:4^1/2^:7^1/2^, indicating a hexagonal lattice (*p*6*mm*) with a lattice parameter *a*_hex_ = 4.15 nm (Fig. 3a and Supplementary Table 1). Upon cooling, the diffractogram was replaced by a set of new sharp peaks when the phase transition occurred at *T* = 78 °C. The new reflections retained the six-fold symmetry with a ratio of 1:3^1/2^:4^1/2^:7^1/2^:9^1/2^:12^1/2^…, whereas a much larger lattice *a*_hex_ = 11.36 nm was adopted, indicating a supertiling (Fig. 3b and Supplementary Table 2). There exist only four sharp reflections when temperature is below 67 °C (ratio 1:2^1/2^:4^1/2^:5^1/2^) corresponding to a typical square lattice (*p*4*mm*) with a smaller lattice parameter *a*_squ_ = 3.29 nm (Fig. 3c and Supplementary Table 3).

### Triangular tiling

**Fig. 5:**
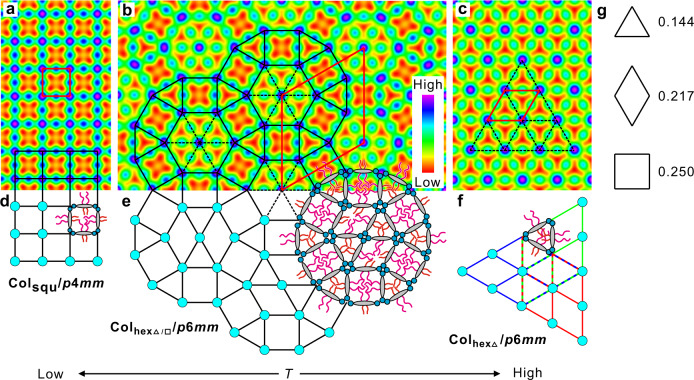
Development of the dodecagonal supertiles at the transition from triangular to square tiling patterns. **a**–**c** ED maps reconstructed from the SAXS diffractograms shown in Fig. 3a–c with models showing the tiling patterns of the rod-like OPE cores in the distinct LC phases (the cells are filled by the lateral alkyl chains), some schematic molecules are added in the models in **d**–**f** to show local molecular orientations; dotted lines indicate defective walls with *n*_wall_ < 1; **f** the 3 overlaid 120° rotated rhombic tilings leading to a defective triangular honeycomb; **e** the tiling with rhombic and triangular tiles forming the middle hexagons in the dodecagonal supertiles (see Supplementary Fig. 10 and the associated Supplementary Discussions 3.1 and 3.2), and **g** the area/circumference ratios of the three discussed polygons. For details of ED reconstruction, see Methods and Supplementary Methods 4.1; alternative phase combinations for the Col_hexΔ/□_/*p*6*mm* phase are shown in the Supplementary Fig. 11.

In the high temperature Col_hex_ phase, the lattice parameter *a*_hex_ = 4.15 nm agrees well with the calculated molecular length between the ends of the glycerol groups, being 4.2 ± 0.2 nm depending on the conformation of the glycerol units (Supplementary Fig. 2). The optical negative spherulitic texture (Fig. 4a) confirms that the intramolecular *π*-conjugation pathway is parallel to the 2D lattice as typical for honeycomb LCs^7^. The reconstructed electron density (ED) map from the diffraction intensities (Supplementary Table 1) shows a simple triangular tiling (Col_hexΔ_ phase, Fig. 5c). The high electron density dots (purple to blue), which form a hexagonal lattice, indicate the positions of the columns of hydrogen-bonded glycerol units while the low-density triangular regions (red to yellow) are composed of aliphatic side chains filling the honeycomb cells. The middle density regions (light blue to green) connecting the glycerol units and separating the alkyl regions represent the prismatic walls of the paralleled OPE cores.

### Dodecagonal supertiles

The ED map of the complex SAXS pattern between 78 and 67 °C reveals a periodic tiling composed of triangular and square cells in a ratio of 8:3 (Col_hexΔ/□_ phase), forming dodecagonal supertiles with a hexagonal core composed of six triangles, being surrounded by a corona formed by alternating triangles and squares (Fig. 5b). The lattice parameter, *a*_hex_ = 11.36 nm ≈ 2.7 *L*_mol_, agrees well with a hexagonal packing of these dodecagonal supertiles with fully overlapping coronas (sketched model in Fig. 5b). To corroborate this complex structure, geometric models of the two hexagonal structures of compound **1** were constructed (Supplementary Figs. 13 and 15) and the diffraction intensities were calculated, which compare well with those observed (Supplementary Tables 5 and 6, for details of the simulations, see Supplementary Methods 4.2). Next, ED maps were reconstructed using the structure factor amplitudes from diffraction intensities and the corresponding phase angles from Fourier analysis of the models, well supporting our proposed structures (see Fig. 5 and the Supplementary Figs. 12 and 16, for details of the ED reconstructions, see Methods and Supplementary Methods 4.1).

The negative birefringence (inset in Fig. 4b) confirms that the OPE cores are aligned on average perpendicular or only slightly tilted to the column long axis, and that a honeycomb structure of the LC phase is retained at the Col_hexΔ_ − Col_hexΔ/□_ transition. The birefringence is almost constant between 110 and 70 °C and does not change at the Col_hexΔ_ − Col_hexΔ/□_ transition at 78 °C (Fig. 4a, b), indicating that the orientation of the rods with respect to the plane of the 2D tiling does not change in this temperature range. At constant side length, the prismatic cells with square cross sectional area provide a 42% larger volume for each chain than available in the triangular cylinders (Fig. 5g), thus reducing the steric frustration present in the triangular tiling. The additional square cells are predominately filled by the larger chains of compound **1**, whereas the shorter chains are preferentially located in the remaining triangular cells (Fig. 5e).

### Square tiling and emergence of tilt

**Fig. 6:**
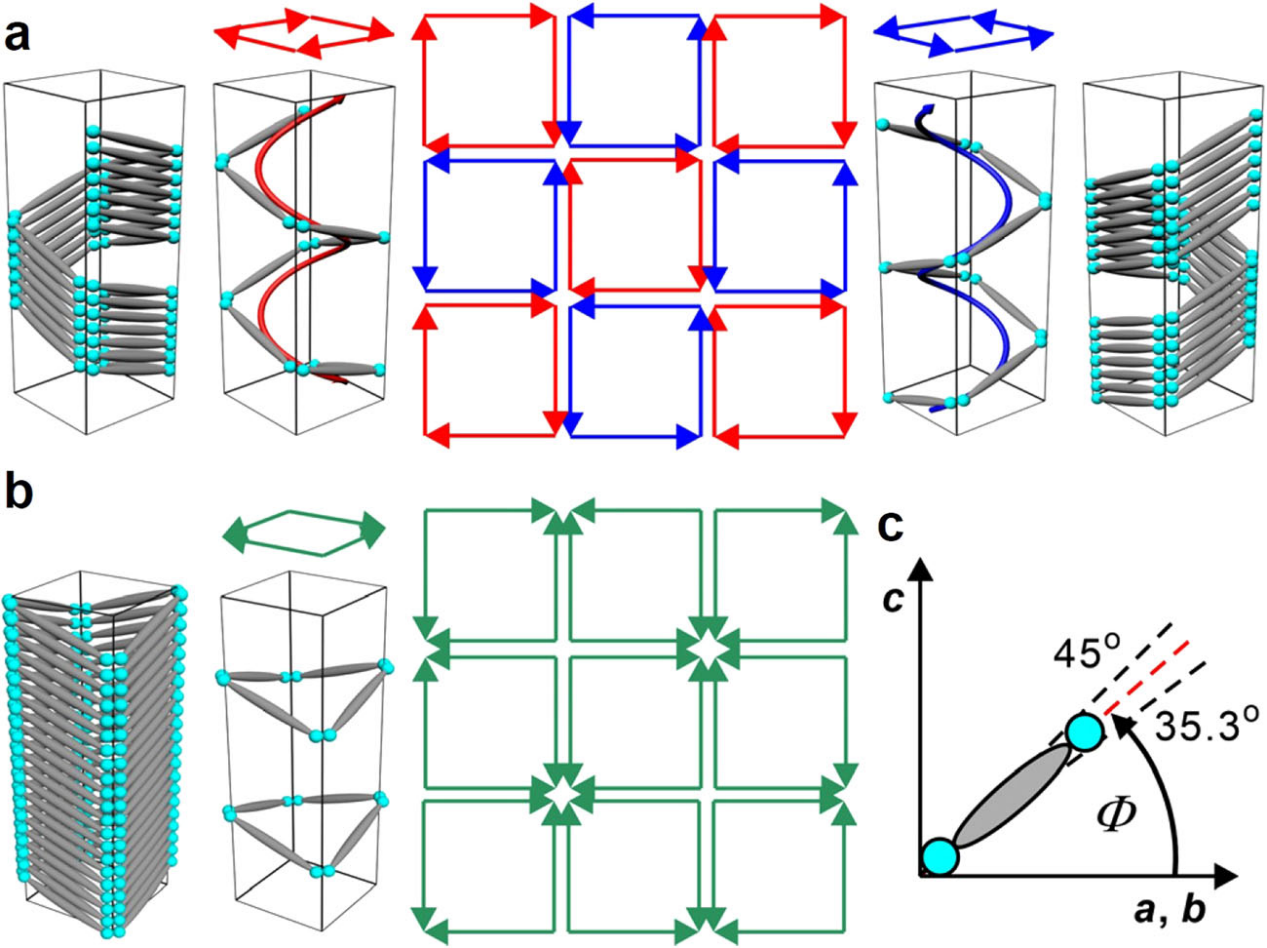
Distinct modes of tilt of the *π*-conjugated rods in the cylinder walls of the Col_squ_^*T*^/*p*4*mm* honeycomb. **a** Synclinic tilt leading to a racemic structure composed of helices with alternating helix sense (red/blue, the arrows directions indicate the tilt directions from bottom to top). **b** Anticlinic tilt leading to achiral cylinders^15^. **c** The definition of the tilt angle *Φ*.

The ED map of the low-temperature phase confirms a square columnar phase (Fig. 5a). However in this Col_squ_/*p*4*mm* phase the lattice parameter (*a*_squ_ = 3.29 nm) is much shorter than the molecular length, indicating a significant tilt of the molecules in the walls (Col_squ_^*T*^/*p*4*mm*, Fig. 6)^15^. This is in line with the observed inversion of birefringence from negative to positive at the Col_hexΔ/□_-Col_squ_^*T*^ transition (see insets in Fig. 4b–d) meaning that at the inversion point the tilt of the OPE rods crosses a critical angle with respect to the plane of the 2D tiling pattern. Using the effective length of the bolaamphiphilic core in the square phase (*L*_mol,eff_ = 4.38 nm)^15^, the estimated tilt (cos*β* = *a*_squ_/*L*_mol,eff_) shortly below the inversion point (at *T* = 67 °C) is about *β* = 41° in the Col_squ_^*T*^/*p*4*mm* phase at *T* = 65 °C. The birefringence only slightly increases upon further cooling and its absolute value remains very small, much smaller than in the Col_hex_ phases (compare Fig. 4b and d). The very weak bluish color in Fig. 4d is in the first order “white” birefringence color range, which appears bluish to the eye because less orange and red color is transmitted, and this is indicative for a very small birefringence. This means that in the square honeycomb structures the tilt is restricted to a certain limiting value close to the critical angle of inversion of birefringence around 40°, which is mainly determined by the prismatic cell volume required by the branched alkyl side chains. As described previously^15^, there are two different modes of tilt of the rods around the square prismatic cells allowing a commensurate packing between them (Fig. 6a, b). If the tilt direction is uniform (synclinic) in the cylinders enclosing the prismatic cells, the molecules assume a helical organization around the prismatic cells and the helix sense alternates from cell to cell, leading to an overall racemic structure (Fig. 6a). If the tilt direction is alternating (anticlinic), all cells are identical and the structure is non-helical and achiral as shown in Fig. 6b. In analogy to results of computer calculations of refractive indices in anticlinic tilted smectic phases formed by chiral and achiral molecules (i.e. with or without heliconical organization of the molecules)^16,17^, the two packing modes can be distinguished by the critical angle at which the birefringence becomes Δ*n* = 0. In the helical arrangement, it takes place at the magic angle (54.7° with respect to the prismatic cell long axis *c*), corresponding to a tilt out of the plane of the 2D lattice by Φ = 35.3° and for the non-helical structure it is at Φ = 45° (Fig. 6c). The calculated angle of Φ = 41° measured at 65 °C, i.e. shortly below the inversion point from negative to positive birefringence, is larger than 35.3°, but has not reached 45°, thus supporting the helical model (Fig. 6a). The synclinic tilt might be favored because it provides an entropic advantage by allowing easier fluctuations of the rods in the honeycomb walls around the prismatic cells.

Remarkably, the transition enthalpy of the Col_hexΔ/□_ − Col_squ_ transition is much larger (10.7 kJ mol^−1^) than the Col_hexΔ_ − Col_hexΔ/□_ transition enthalpy (0.8 kJ mol^−^^1^, Fig. 1b and Table 1). The fundamental honeycomb structure is largely retained at the transition between the two hexagonal phases and the WAXS also does not change, whereas the transition to the square honeycomb is accompanied by the development of a tilt and a narrowing of the wide-angle scattering (Fig. 3d–f and Supplementary Fig. 3). This narrowing is similar to the transition from a LC to a hexatic (for example, HexF)^18^ phase, indicating that the packing density increases. Because there is only a single wide-angle scattering without any additional scattering for a *π*–*π* stacking distance, the packing of the relatively electron rich polyaromatic rods is likely to assume an edge-to-face stacking^19–21^. The developing dense chain- and core-packing leads to the significant enthalpy change at the Col_hexΔ/□_ − Col_squ_^*T*^ transition. The electrostatic core–core interactions are known to support a displacement between the interacting *π*-systems, thus supporting the development of a tilt of the cores in the honeycomb walls. However, the major driving force for tilt arises from the difference between the large available cell volume in the square cells and the smaller chain volume, which is reduced by tilting of the rod-like units in the walls around the square prismatic cells. The emerging tilt is additionally promoted by the denser alkyl chain packing at reduced temperature, further reducing the space required by these chains, and by the alkyl chain stiffening, which supports an organization of the alkyl chains parallel to each other and parallel to the column long axis. In order to maximize orientational order and dispersion interactions by minimizing the excluded volume, the OPE cores also tend to align parallel to the chains, which additionally supports the tilt^15^. Thus, there are multiple factors contributing to the development of tilt, which enhance each other and therefore the Col_hexΔ/□_ − Col_squ_^*T*^ transition is likely to be highly cooperative in nature^22,23^. Because for compound **1** the main orientation of the alkyl chains is parallel to the prismatic cell long axis, the expansion of the alkyl chains takes place perpendicular to the plane of the 2D lattice, and therefore, the lattice shrinks with decreasing temperature, whereas in conventional columnar and lamellar LCs, having the chains predominately aligned parallel to the lattice direction(s), the opposite is observed—chain stiffening leads to an expansion of the lattice.

### Defective triangular tilings and randomized rhomb tilings

Although in the square phase, adjusting the larger free volume in the square cells to the smaller volume of the mixed alkyl chains is achieved by reduction of the cell size by tilting of the bricks in the walls, in the Col_hexΔ_ phase the mixed lateral chains are too big for the triangular prismatic cells. Calculation of the number of molecules in each hexagonal unit cell of the Col_hexΔ_ phase with a fixed hypothetical height of *h* = 0.45 nm^24^ by two different methods (Supplementary Tables 7 and 10 and Supplementary Methods 4.3) leads to *n*_cell_ = 2.5 molecules (Table 1). This would mean that in the lateral cross-section of the walls of the triangular honeycomb there is on average a bit less than exactly one OPE core (*n*_wall_ = *n*_cell_/3 = 0.83). It indicates a somewhat defective triangular honeycomb with holes in the walls, which is considered to be formed by overlapping rhombic cells from different stratum of the columns having three distinct directions (green, red and blue in Fig. 5f)^11^. This steric frustration is partly solved in the Col_hexΔ/□_ phase by formation of additional square cells alternating with the triangular. In this way, the longer and shorter chains can segregate and accumulate in the larger square and much smaller triangular cells, respectively, removing the tilt in the square cells and the overcrowding in the triangular cells of the coronas (Fig. 5e). However, in the hexagonal clusters of triangular cells in the centers of the dodecagonal supertiles some steric frustration is retained, because the long chains of the molecules forming the walls between two triangular cells cannot escape into a square cell, and thus have to be incorporated into the smaller triangular cells (Fig. 5e). To avoid overcrowding within these triangular cells, the number of molecules in these cell has to be reduced, which is indicated in the ED map (Fig. 5b) by a reduced electron density in the walls separating adjacent triangular cells, and by a surprisingly small size of the high electron density dot of the higher valence six-fold junction in the middle of the hexagons if compared to the lower valence five-fold junctions in the coronas. A local structure formed by two alternating rhombic and triangular cells, as shown in Fig. 5e^11,25^, would allow the segregation of the long chains into the larger rhombic cells and thus removes the overcrowding in the remaining triangular cells (for more details, see Supplementary Fig. 10 and Supplementary Discussion 3.2). The rhomb orientation in the hexagons is randomized, i.e. the orientation of the rhombs is only weakly coupled along as well as between the supertiles and thus time and space averaging of the rhomb orientation retains the hexagonal lattice (Fig. 5e). This adjustment of chain and cell volume would not be possible in the alternative *p*4*gm* lattice (Fig. 1e) composed of squares and pairs of triangles, where removing the walls between adjacent triangles would lead to rhombs surrounded exclusively by square cells, i.e. the self-sorting of long and short chains would be reduced. The entropically and energetically stabilized local disorder in the dodecagonal supertiles is assumed to additionally stabilize these supertiles.

### Effect of core fluorination

The peripheral core fluorinated compound **1F** shows the same dodecagonal superlattice in an even wider temperature range together with the triangular honeycomb, whereas the square honeycomb and the cubic phase were removed (Table 1, Supplementary Figs. 1b, 4–6, Supplementary Tables 1 and 4 and Supplementary Methods 4.3). This shows that core fluorination can stabilize the dodecagonal superstructure. Probably, the fluorines can distort the edge-to-face packing of the polyaromatic cores, and thus disfavor the development of the tilt in the square cells. These results are only preliminary, a deeper understanding of the steric and electronic effects of core fluorination on soft self-assembly requires additional structural variations by changing the number and positions of the fluorine substituents.

### Effect of side-chain distribution

Compound **2** has the same total chain volume and the same length of the branches (2 × C_8_H_17_ + 2 × C_22_H_45_) as compounds **1** and **1F**, but chains with different length are combined (“mixed”) in the identical lateral chains at both sides. This compound does not form the Col_hexΔ/□_ phase and only the triangular honeycomb is observed besides the cubic phase (Table 1, Supplementary Figs. 1c, 7–9, Supplementary Table 1 and Supplementary Methods 4.3). This suggests that the segregation of long and short chains into the larger square and smaller triangular cells is essential for the formation of the dodecagonal tiling and provides a unique example for the self-sorting of “chemical identical” alkyl chain, solely based on a different volume. The transition to the square lattice with tilted molecules might be suppressed by the reduced capability of the “mixed” side chains to assume a sufficiently dense packing.

## Discussion

A nanoscale periodic dodecagonal supertile motif (Fig. 5b) was produced in the fluid LC state by the bulk self-assembly of star-like bolapolyphiles with two sticky ends. Similar tiling patterns have previously only been obtained as solid state 2D-nets on surfaces^26–28^ and by DNA-nanotechnolgy^29^ and were found in layers of periodic and quasiperiodic sphere packings^30,31^, including tetrahedrally closed-packed phases in the metallic superalloys^32^, the Frank–Kasper phases^33^ and dodecagonal quasicrystals^34^. In contrast, the structures reported here, represent unique fluids composed of self-assembled polygonal columns, thus representing ~10-nm-scale analogues of related polymer morphologies occurring on a much larger length scale^35–37^. Aside from allowing much smaller patterns, the unique features of these self-assembled structures of low molecular weight molecules are the high dynamics and the relatively low viscosity if compared with the related morphologies of block copolymers. Therefore, the distinct tiling patterns reported here represent thermodynamically stable phases, which form spontaneously and transform into one another at distinct well defined phase transitions, usually without significant hysteresis. In this respect these LC phases are distinct from polymer morphologies, representing solid state structures, usually growing from solutions by slow evaporation with structures being strongly affected by surface interactions^31,32^. The complex LC phases reported here form independent on a surface pattern or surface lattice and other external stimuli (shear forces, external electric or magnetic fields), though these stimuli can be used to align the LC phases on a macroscopic scale.

Overall, this work shows that precise design of low molecular mass tectons can lead to amazingly complex superstructures, due to a combination of steric and geometric frustration, occurring at the transition between two simpler modes of self-assembly, resembling regular tilings. Remarkably, the formation of the reported complex fluid with dodecagonal supertiles is obviously supported by the segregation of longer from shorter “chemical identical” alkyl chains. This periodic supertiling pattern can be considered as a close approximant of the still elusive columnar LQCs with dodecagonal symmetry with a slightly reduced triangle to square ratio of 6.93:3^36^.

## Methods

### Synthesis

**Fig. 7:**
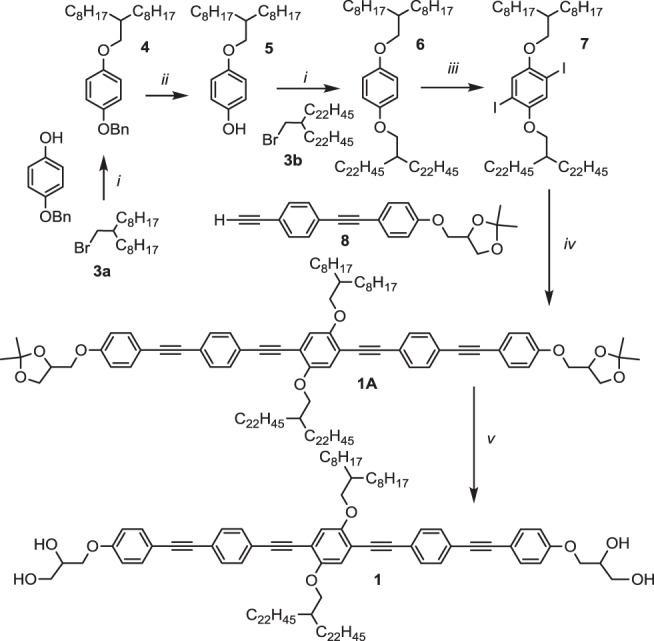
Synthesis of compound 1. Reagents and conditions: i DMF, K_2_CO_3_, 120 °C, 12 h, 44%; ii H_2_, Pd(OH)_2_, 1,4-dioxane, 50 °C, 12 h, 92 %; iii I_2_, PhI(OCOCF_3_)_2_, CH_2_Cl_2_, 20 °C, 12 h, 33 %^38^; iv Pd(PPh_3_)_4_, CuI, Et_3_N, 100 °C, 6 h, 95 %; v- PPTS, MeOH, THF, 50 °C, 12 h, 70%.

Compounds **1** and **2** were synthesized in-house according to the synthetic procedure shown in Fig. 7 for compound **1** as example. The synthesis of dialkoxy-2,5-diiodobenzene **13** having two identical chains with different branches (C_8_H_17_ + C_22_H_45_), replacing compound **7** in the synthesis of **2**, is shown in the Supplementary Fig. 17 and the Supplementary Fig. 18 shows the synthesis of compound **1F**. Details of the experimental procedures and the analytical data of the compounds **1**, **1F** and **2** are described in the accompanying Supplementary Methods 4.4 and their NMR spectra are shown in the Supplementary Figs. 19–25.

### Optical and calorimetric investigations

Phase transitions were determined by polarizing microscopy (Leica DMR XP) in conjunction with a heating stage (FP 82 HT, Mettler) and controller (FP 90, Mettler), and by DSC (DSC-7, Perkin Elmer) at heating/cooling rates of 10 K min^−1^ (peak temperatures). Optical investigation was carried out under equilibrium conditions between glass slides, which were used without further treatment; sample thickness was ~15 μm. A full-wavelength retardation plate was used to determine the sign of birefringence.

### In-house X-ray scattering on powder-like samples

X-ray investigations on powder-like samples were carried out at Cu-Kα line (*λ* = 1.54 Å) using standard Coolidge tube source with a Ni-filter. Samples were prepared in the isotropic state on a glass plate. The sample was cooled (rate: 5 K min^−1^) to the measuring temperature. The samples were held on a temperature-controlled heating stage and the diffraction patterns were recorded with a 2D detector (Vantec 500, Bruker); exposure time was 15–30 min. For the WAXS measurement, the distance between the sample and the detector defined to be 9.0 cm; for SAXS measurement, the distance is 26.8 cm. As result a XRD pattern is obtained, which is transformed in a 1D plot by using GADDS over the full *χ*-range.

### Synchrotron X-ray diffraction and electron density reconstruction

SAXS experiments were recorded at Beamline BL16B1 at Shanghai Synchrotron Radiation Facility, SSRF. Samples were held in evacuated 1 mm capillaries. A modified Linkam hot stage with a thermal stability within 0.2 °C was used, with a hole for the capillary drilled through the silver heating block and mica windows attached to it on each side. A MarCCD detector was used. *q* calibration and linearization were verified using several orders of layer reflections from silver behemate and a series of *n*-alkanes. The measurement of the positions and intensities of the diffraction peaks is carried out using Galactic PeakSolve^TM^ program, where experimental diffractograms are fitted using Gaussian shaped peaks. The diffraction peaks are indexed on the basis of their peak positions, and the lattice parameters and the space groups are subsequently determined. Once the diffraction intensities are measured and the corresponding plane group determined, electron density maps can be reconstructed, on the basis of the general formula

1 $${\it{E}}\left( {{\it{xy}}} \right) = \mathop {\sum}\limits_{{\it{hk}}} {{\it{F}}\left( {{\it{hk}}} \right){\mathrm{exp}}\left[ {{\mathrm{i}}2{\uppi}\left( {{\it{hx}} + {\it{ky}}} \right)} \right]}.$$

Here *F*(*hk*) is the structure factor of a diffraction peak with index (*hk*). It is normally a complex number and the experimentally observed diffraction intensity.

2 $${\it{I}}\left( {{\it{hk}}} \right) = {{K}} \cdot {\it{F}}\left( {{\it{hk}}} \right) \cdot {\it{F}} \ast \left( {{\it{hk}}} \right) = {{K}} \cdot \left| {{\it{F}}\left( {{\it{hk}}} \right)} \right|^2.$$

Here *K* is a constant related to the sample volume, incident beam intensity etc. In this paper, we are only interested in the relative electron densities, hence this constant is simply taken to be 1. Thus, the electron density.

3 $${\it{E}}\left( {{\it{xy}}} \right) = \mathop {\sum}\limits_{{\it{hk}}} {{\mathrm{sqrt}}\left[ {{\it{I}}\left( {{\it{hk}}} \right)} \right]{\mathrm{exp}}\left[ {{\mathrm{i}}2{\uppi}\left( {{\it{hx}} + {\it{ky}}} \right) + \phi _{{\it{hk}}}} \right]}.$$

As the observed diffraction intensity *I*(*hk*) is only related to the amplitude of the structure factor |*F*(*hk*)|, the information about the phase of *F*(*hk*), *ϕ*_*hk*_, cannot be determined directly from experiment. However, the problem is much simplified when the structure of the ordered phase is centrosymmetric, and hence the structure factor *F*(*hk*) is always real and *ϕ*_*hk*_ is either 0 or *π*.

This makes it possible for a trial-and-error approach, where candidate electron density maps are reconstructed for all possible phase combinations, and the “correct” phase combination is then selected on the merit of the maps, helped by prior physical and chemical knowledge of the system. This is especially useful for the study of nanostructures, where normally only a limited number of diffraction peaks are observed. For more details, see the Supplementary Methods 4.1.

### Calculation of structural data and development of structural models

Based on the SAXS and WAXS data (Supplementary Tables 1–3) structural models were developed for the distinct LC phases as described in the Supplementary Methods 4.3.

### Simulation of XRD intensities

Simulation of the SAXS patterns of the Col_hex_ phases of compound **1** was conducted using geometric 2D models (Supplementary Figs. 13 and 15, Supplementary Tables 5 and 6) as described in the Supplementary Methods 4.2.

## Supplementary information

Supplementary Information

## Data Availability

All data are available from the authors upon reasonable request.
