# Supplementary material for: A periodic dodecagonal supertiling by self-assembly of star-shaped molecules in the liquid crystalline state
Source: Commun Chem. 2020 Jun 1;3:70. doi: 10.1038/s42004-020-0314-1 (PMC9814142; doi:10.1038/s42004-020-0314-1)
Supplement: Supplementary file 1 — Supplementary Information [file 42004_2020_314_MOESM1_ESM.pdf]

Supplementary Information

A Periodic Dodecagonal Supertiling by Self-assembly of Star-shaped Molecules in the Liquid Crystalline State

Marco Poppe[a, #], Changlong Chen[b, #], Silvio Poppe[a], Feng Liu[b, *], Carsten Tschierske[a, *]

[a] Institute of Chemistry, Martin-Luther-University Halle-Wittenberg, Kurt-Mothes-Straße 2, 06120 Halle (Germany)
E-mail: carsten.tschierske@chemie.uni-halle.de

[b] State Key Laboratory for Mechanical Behaviour of Materials, Shaanxi International Research Center for Soft Matter, Xi'an Jiaotong University, Xi'an 710049 (P. R. China)
E-mail: feng.liu@xjtu.edu.cn

1. Supplementary Figures

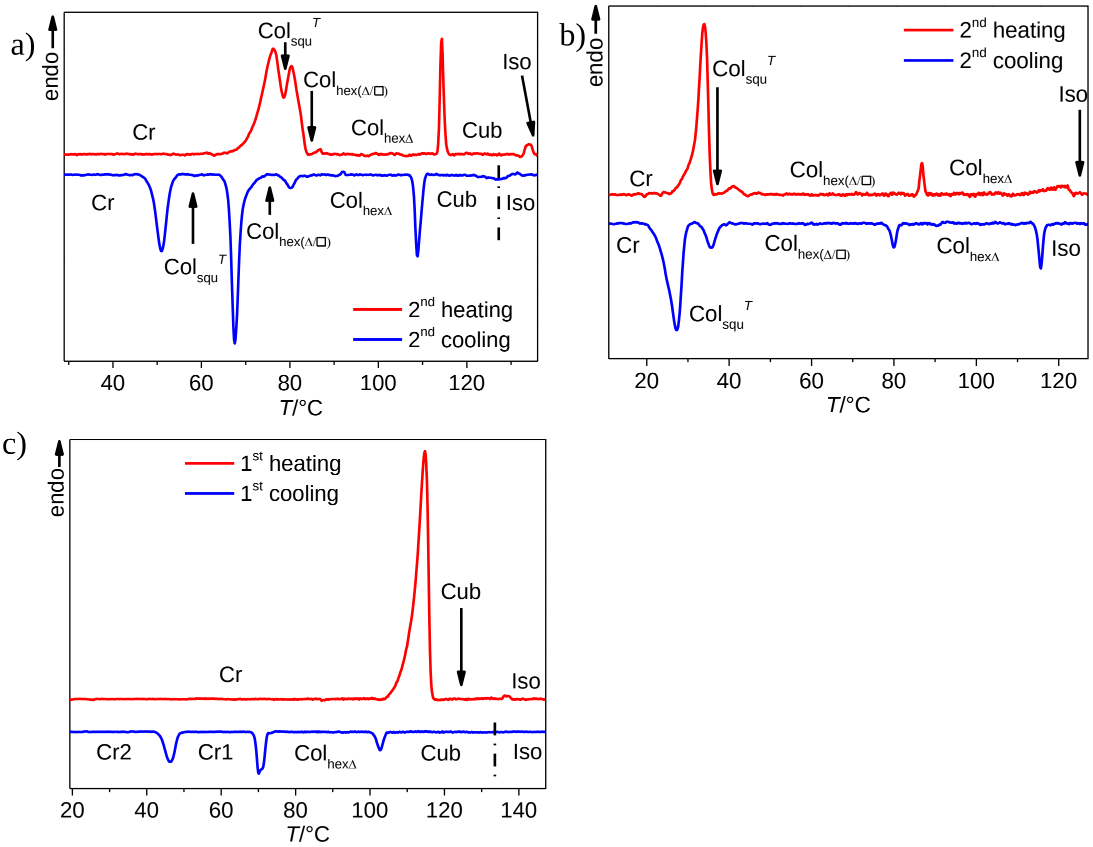

Supplementary Figure 1. Representative DSC heating and cooling traces of compounds a) **1** (heating and cooling cycles measured between 20 ℃ and 140 ℃, with crystallization); b) **1F** and c) **2** recorded at 10 K min^{-1}.

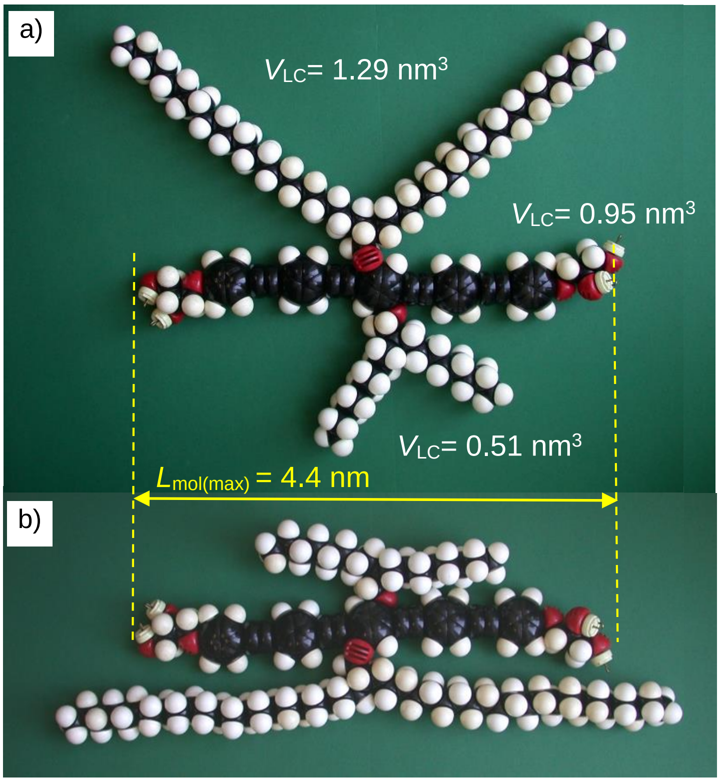

Supplementary Figure 2. Space filling (CPK) models showing the star-shaped bolaamphiphile **1** (a) in a star-like conformation and (b) with parallel alignment of cores and chains; the volumes calculated according to Immirzis increments are: core unit: V_{cr} = 0.85 nm^3, long chains 1.15 nm^3, short chain 0.45 nm^3 and with correction for the reduced packing density in the LC state: (V_{LC} = V_{cr} / 0.89): V_{LC} = 0.95, 1.29, 0.51 nm^3, respectively.

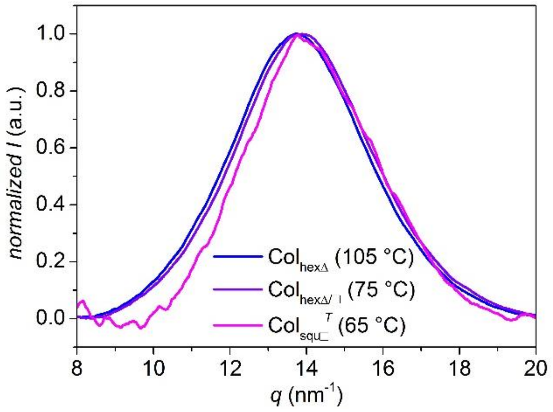

Supplementary Figure 3. WAXS diffractograms of the different mesophases formed by **1** show all wide angle scatterings after smoothing in one diagram.

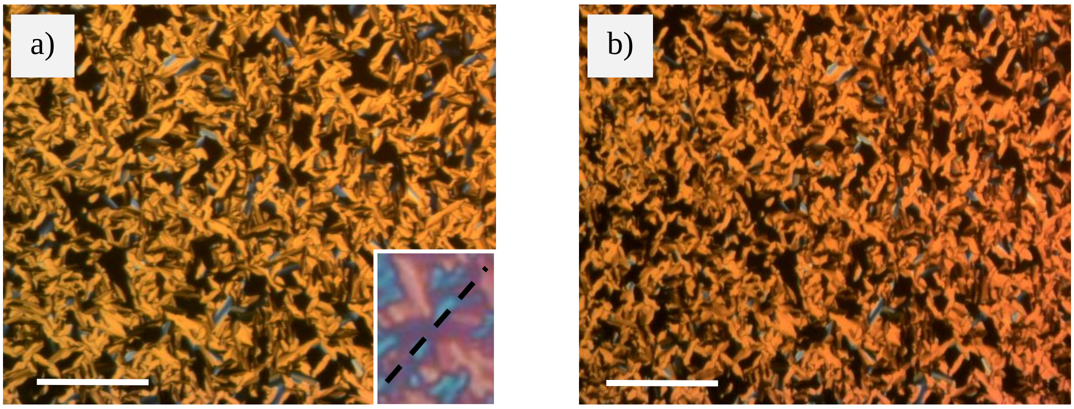

Supplementary Figure 4. POM textures of compound **1F** on cooling from the isotropic state. a) optical negative spherulitic texture of the simple Col$_{hex\Delta}$ LC phase at 100 ℃ and b) texture of the complex Col$_{hex\Delta/\square}$ LC phase at 60 ℃; the scale bar is 200 μm.

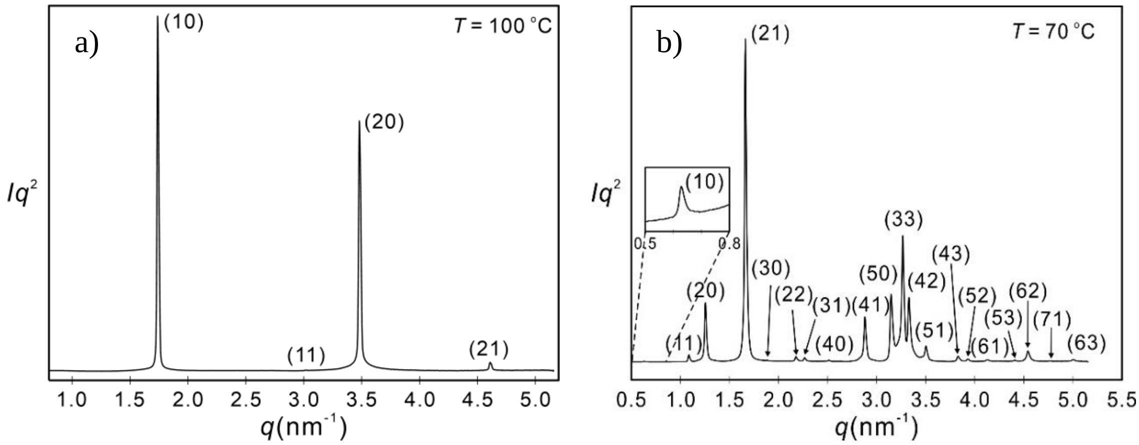

Supplementary Figure 5. SAXS diffractograms of compound **1F** a) Col$_{hex\Delta}$/*p*6*mm* and b) Col$_{hex\Delta/\square}$/*p*6*mm* phase at the given temperatures.

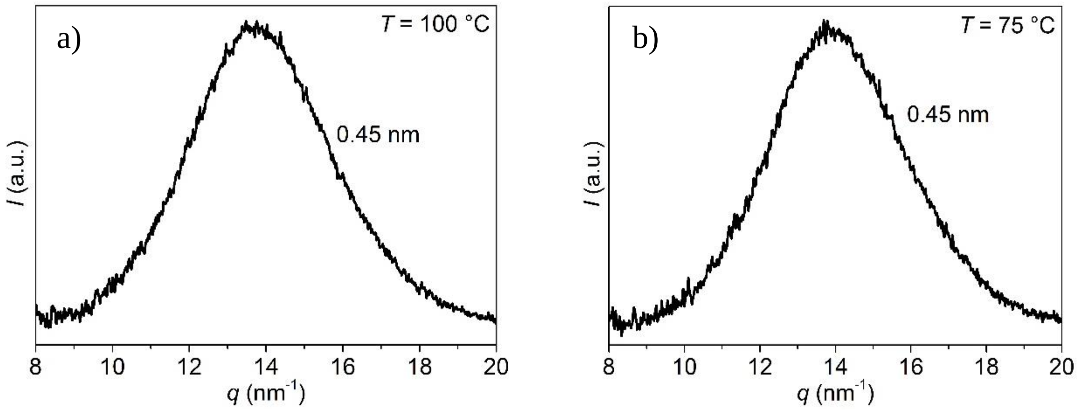

Supplementary Figure 6. WAXS diffractograms of the different mesophases formed by **1F**. a) simple Col$_{hex\Delta}$/*p*6*mm* phase at 100 ℃ (d = 0.45 nm) and b) complex Col$_{hex\Delta/\square}$/*p*6*mm* phase at 75 ℃ (d = 0.45 nm).

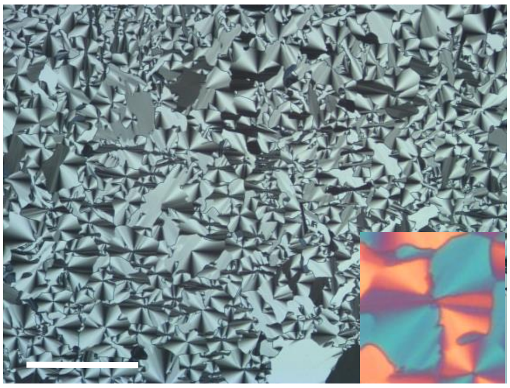

Supplementary Figure 7. Optical negative spherulitic texture of the simple $Col_{hex\Delta}$ LC phase of compound **2** at 90 ℃; the scale bar is 200 μm.

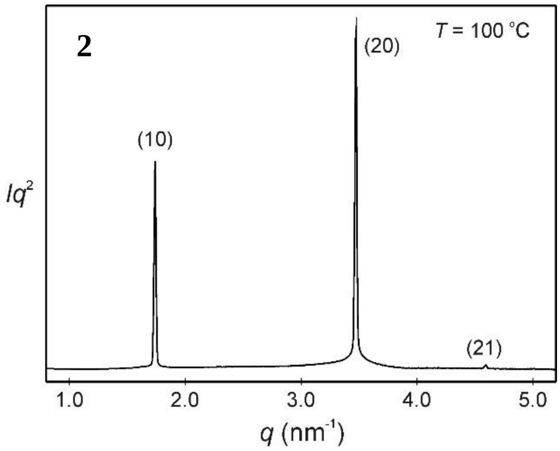

Supplementary Figure 8. SAXS diffractogram of the $Col_{hex\Delta}$/*p*6*mm* phases of compound **2** at 100 ℃.

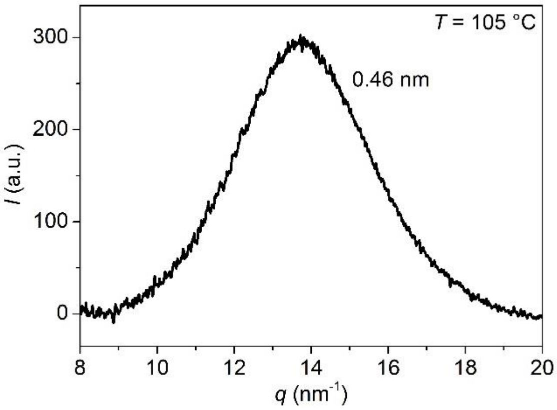

Supplementary Figure 9. WAXS diffractogram of the simple $Col_{hex\Delta}$/*p*6*mm* phase of compound **2** at 105 °C.

2. Supplementary Tables

Supplementary Table 1. Experimental and calculated *d*-spacings of the observed SAXS reflections of the simple hexagonal phases ($Col_{hex\Delta}$/*p*6*mm*). All intensities values are Lorentz and multiplicity corrected.

Comp.	(*hk*)	$d_{obs.}$ (nm)	$d_{calc.}$ (nm)	*intensity*	*phase*	a_{hex}/*nm* (*T*/°C)
**1**	(10)	3.60	3.60	66.8	0	4.15 (105)
	(20)	1.80	1.80	100.0	0	
	(21)	1.36	1.36	0.3	π	
**1F**	(10)	3.61	3.60	100.0	0	4.16 (100)
	(11)	2.08	2.08	0.2	π	
	(20)	1.80	1.80	85.6	0	
	(21)	1.36	1.36	1.5	π	
**2**	(10)	3.61	3.61	59.8	0	4.17 (100)
	(20)	1.81	1.80	100.0	0	
	(21)	1.37	1.36	0.6	π	

Supplementary Table 2. Experimental and calculated *d*-spacings of the observed SAXS reflections of the $Col_{hex\Delta/\square}$/*p*6*mm* phase of compound **1** at 75 ℃. All intensities values are Lorentz corrected with correction for multiplicity.

(*hk*)	$d_{obs.}$ (nm)	$d_{calc.}$ (nm)	*intensity*	*phase*
(10)	9.82	9.83	0.2	0
(11)	5.68	5.68	3.2	0
(20)	4.92	4.92	41.6	π
(21)	3.72	3.72	73.6	0
(30)	3.29	3.28	2.4	π
(22)	2.84	2.84	7.9	π
(31)	2.73	2.73	2.0	0
(40)	2.46	2.46	2.8	π
(41)	2.15	2.15	16.8	π
(50)	1.97	1.97	42.6	0
(33)	1.90	1.89	100.0	0
(42)	1.86	1.86	33.4	0
(51)	1.77	1.77	6.4	π
(43)	1.62	1.62	1.2	π
a_{hex} = 11.36 nm				

Supplementary Table 3. Experimental and calculated *d*-spacings of the observed SAXS reflections of the tilted square phase of compound **1** at 65 ℃. All intensities values are Lorentz and multiplicity corrected.

(*hk*)	$d_{obs.}$ (nm)	$d_{calc.}$ (nm)	*intensity*	*phase*	a_{squ}/nm
(10)	3.29	3.29	100.0	0	3.29
(11)	2.33	2.32	10.1	0	
(20)	1.65	1.64	80.4	0	
(21)	1.47	1.47	0.6	π	

Supplementary Table 4. Experimental and calculated *d*-spacings, relative integrated intensities, and phases used in the reconstruction of electron densities for the $Col_{hex\Delta/\square}/p6mm$ phase of compound **1F** at 80 ℃. All intensities values are Lorentz and multiplicity corrected.

(*hk*)	$d_{obs.}$ (nm)	$d_{calc.}$ (nm)	*intensity*	*phase*
(10)	9.97	9.99	0.2	0
(11)	5.77	5.77	3.8	0
(20)	5.00	5.00	34.3	π
(21)	3.78	3.78	100.0	0
(30)	3.33	3.33	0.2	π
(22)	2.88	2.89	2.5	π
(31)	2.77	2.77	0.9	0
(40)	2.50	2.50	0.6	π
(41)	2.18	2.18	14.2	π
(50)	1.99	2.00	44.4	0
(33)	1.92	1.92	71.0	0
(42)	1.89	1.89	14.6	0
(51)	1.79	1.79	4.5	π
(43)	1.64	1.64	2.0	/
(52)	1.60	1.60	0.9	/
(61)	1.52	1.52	0.7	/
(53)	1.42	1.43	0.3	/
(62)	1.38	1.39	4.7	/
(71)	1.32	1.32	0.2	/
(63)	1.26	1.26	0.8	/
a_{hex} = 11.54 nm				

3. Supplementary Discussion

3.1 Structure of the $Col_{hex\Delta}$/*p*6*mm* phase. Assuming a triangular honeycomb (Δ in Supplementary Tables 7, 10 and 11) would lead to n_{wall} = 0.83-0.84 which is significantly less than the usually observed values around n_{wall} = 1.1-1.2, typically observed for this kind of molecules with two lateral chains.[1] This indicates a strongly defective triangular honeycomb. A value of n_{wall} = 1.25-1.26 was calculated for the alternative structure formed by rhombic cells being orientationally disordered by 60 ° flips along the columns as well as between the columns (◊ in Supplementary Tables 7, 10 and 11). Therefore, it is assumed that in fact the Col_{hex} phase basically represents a random rhomb tiling, overall leading to a defective triangular honeycomb, as previously proposed for oligothiophene based bolaamphiphiles.[2]

3.2 Structure of the possible structure of the $Col_{hex\Delta/\square}$/*p*6*mm* phase with overlapping dodecagonal tiles. There are in principle three possible models for the giant hexagonal phase formed by dodecagonal supertiles ($Col_{hex\Delta/\square}$), differing in the mode of division of the central hexagon (Supplementary Figure 10). The first model ($Col_{hex\Delta/\square}$(Δ)) involves a hexagon formed by six triangles. In this case the longer chains of the molecules in the walls between two adjacent triangles have no way to escape into a larger square cell, making this structure unlikely. This is in line with the electron density maps showing a reduced electron density of the central high ED dots in the middle of the hexagons and a reduced electron density modulation within the hexagons. In addition, the blue dots of the 6-fold junctions in the middle of the honeycomb walls between adjacent triangular cells have almost the same size and ED compared to the only 5-fold junctions located between the squares and triangles in the coronas. This suggests that either these walls are significantly narrower or not all triangles are separated by walls. The second model ($Col_{hex\Delta/\square}$(Δ/◊)) involves two triangular cells separated by two rhombic cells. This would allow an escape of the longer chains into the larger rhombic cells. Finally there is the option that three rhombs divide the hexagons ($Col_{hex\Delta/\square}$(◊)). In the two latter cases the orientation of the rhombs is again rotationally disordered by 60 ° angles along the column long axis as well as between the columns, so that they always appear like tilings by six hexagons, thus retaining the hexagonal *p*6*mm* symmetry. Calculation of the number of molecules per unit cell with h = 0.45 nm leads to 18.3-18.5 molecules which are distributed between 18, 16 and 15 walls, respectively. For the triangular model $Col_{hex\Delta/\square}$(Δ) the average number n_{wall} in the whole structure is n_{wall} = 1.03 which is relatively small. If n_{wall} = 1.2 is assumed for the walls in the coronas, then 4 molecules remain to be distributed between the 6 walls in the central hexagon,

i.e. n_{wall} of these walls between the triangles would be even only 0.67. In contrast n_{wall} = 1.0 was obtained for the four walls in the hexagons of the $Col_{hex\Delta/\square}(\Delta/\lozenge)$ model and n_{wall} = 1.33 was obtained for the three walls in the hexagons of the $Col_{hex\Delta/\square}(\lozenge)$ structure. Hence, a structure composed of 2 rhombic and 2 triangular cells, appears to be the most likely structure. As the electron density (ED) of the middle high ED dot in the hexagons is only slightly smaller than those of the 5-fold nodes (with valency v = 5) of the other glycerol columns in the corona (see Supplementary Figure 12), a structure with four-fold nodes (v = 4) of the middle glycerol columns, as found in the $Col_{hex\Delta/\square}(\Delta/\lozenge)$ model (having 2 fourfold and 4 fivefold nodes in the corona, v = 4.7), appears to be the most likely. For the $Col_{hex\Delta/\square}(\Delta)$ structure with sixfold nodes (v = 6) this central dot would be expected to be larger compared to the dots in the corona with v = 5, and for the $Col_{hex\Delta/\square}(\lozenge)$ structure with a threefold node in the middle of the hexagon (v = 3) it is expected to be much lower than in the nodes of the corona (3 fourfold and 3 fivefold nodes, v = 4.5).

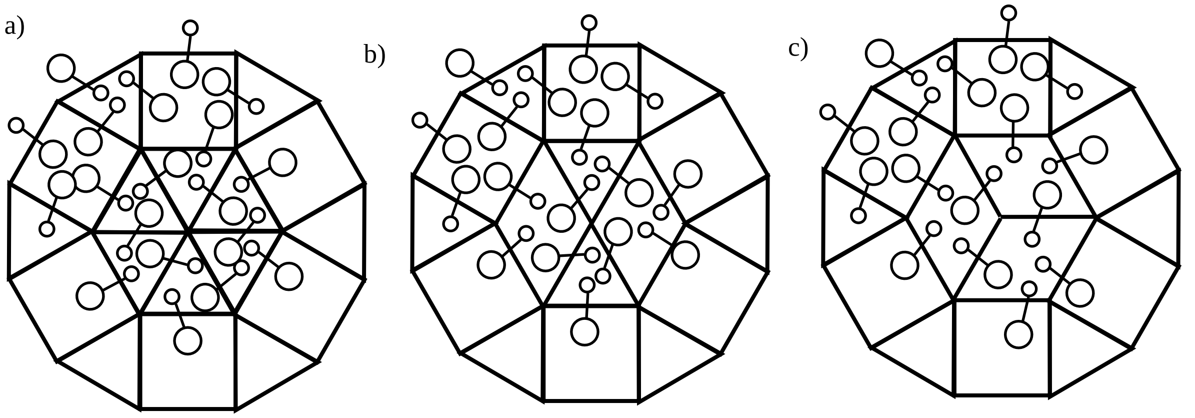

$Col_{hex\Delta/\square}(\Delta)$ (6/5) | $Col_{hex\Delta/\square}(\Delta/\lozenge)$ (4/4.7) | $Col_{hex\Delta/\square}(\lozenge)$ (3/4.5)

Supplementary Figure 10. Lateral chain distributions in the distinct models of the $Col_{hex\Delta/\square}$ phase with the ratio of the valences of the nodes in the central hexagons and in the corona (bottom line).

Similar conclusions could be drawn by estimation using volumes: In the $Col_{hex\Delta/\square}(\Delta)$ structure each of the triangles in the hexagon must accommodate one large and two small chains (Supplementary Figure 10a), whereas the triangular cells in the hexagons of the $Col_{hex\Delta/\square}(\Delta/\lozenge)$ structure accommodate only three small chains (Supplementary Figure 10b). Assuming n_{wall} = 1, this would mean that either 2.43 or 1.52 nm^3 would be required by the chains in each cell providing $3.45 - (3 \times 0.95/2) = 3.45 - 1.43 = 2.02$ nm^3. Only the triangular cells in the $Col_{hex\Delta/\square}(\Delta/\lozenge)$ structure can accommodate the chain volume without reducing n_{wall} below the

critical value of n_{wall} = 1.0. For the rhombic cells the available volume is 6.9 - (2 × 0.95) = 4.0 nm^3. In the rhombic cells the volume is filled by two long and two short chains, providing 2.57 + 1.14 = 3.71 nm^3, fitting well into the available rhombic cell volume, leaving some free space which can be compensated by increasing n_{wall} to a reasonable value of n_{wall} = 1.1. Thus, only in the $Col_{hex\Delta/\square}$(Δ/◊) case the triangular cells can accommodate the chain volume. In the case of the $Col_{hex\Delta/\square}$(◊) structure each rhomb would be filled by one large and three short chains 1.29 + 1.52 = 2.81 nm^3 (Supplementary Figure 10c). Thus only a part of the available space of 4.0 nm^3 (6.9 - 2 × 0.95 nm^3) would be filled, requiring an increase of n_{wall} by 4.0/2.81 = 1.4 which is a bit too large for molecules with branched chains at opposite sides (n_{wall} is usually limited to 1.1-1.2 and was found to be 1.19 in the Col_{squ}^{T} phase of **1**); therefore, we prefer the $Col_{hex\Delta/\square}$(Δ/◊) model.

4. Supplementary Methods

4.1 Reconstruction of electron density maps

For centrosymmetric structures, the phases of the Bragg peaks can only be 0 or π. As there exits only limited number of peaks for the liquid crystal phases, it is convenient to use adopt the enumeration method in choosing the right phase combination. Since the structure of the high-temperature hexagonal structure and the low-temperature square structure are relatively simple, here we take the middle-temperature giant hexagonal phase as an example to show how the proper phase combination is adopted.

Since the strong peaks dominate the main feature of the electron density maps, the strongest two peaks of compounds **1** and **1F**, i.e. (21) and (33), are used for the reconstruction of the electron density maps. The phase combination ππ and π0 are simple reverses of 00 and 0π respectively. According to the molecular structures, the glycerol units tend to form high-density regions with middle-density rigid cores being connecting rods. Thus, only the phase combination 00 meets the geometrical constraints for the molecular length and lattice parameter, and the segregation for different components of the molecules, as shown in Supplementary Figure 11.

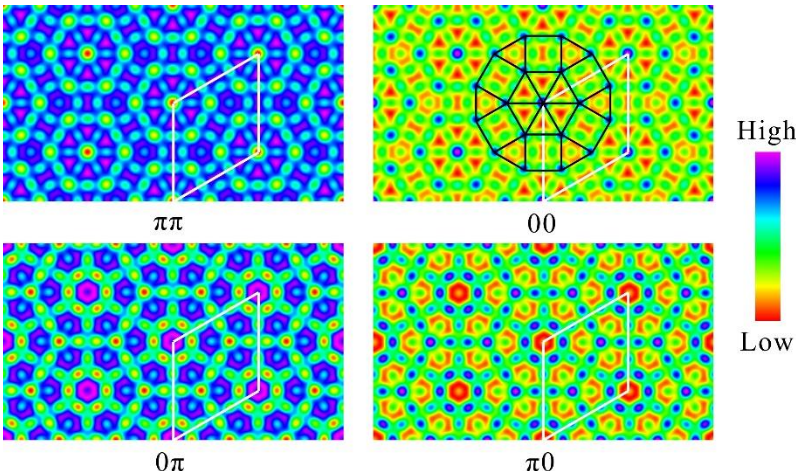

Supplementary Figure 11. Reconstructed electron density map of the $Col_{hex\Delta/\square}/p6mm$ phase of compound **1** using the strongest two peaks (21) and (33). The white lines show a unit cell and the black lines show the tiling pattern.

After determining the main feature, it is straightforward to choose the proper phases for the rest peaks. There are two main principles to follow. First, the resolution of the electron density map will be improved as more peaks are used in the reconstruction, meaning that the segregation of the different components will be more obvious. Furthermore, low-density regions should be

uniform since the alkyl chains are nearly in isotropic state. The final electron density map is shown in Supplementary Figure 12 and the phases are listed in Supplementary Table 2.

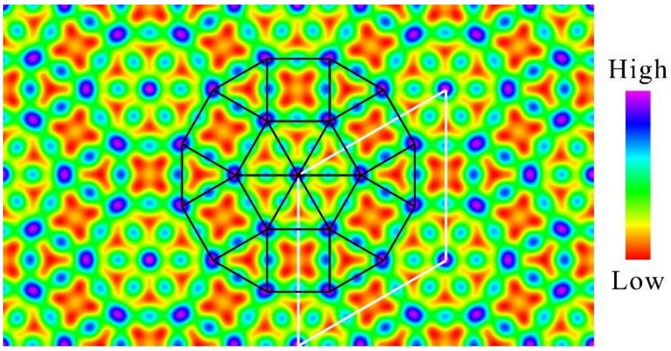

Supplementary Figure 12. Final electron density map of the Col$_{hex\Delta/\square}$/*p*6*mm* phase of compound **1** using all the reflection peaks. The white lines show a unit cell and the black lines show the tiling pattern.

4.2 Simulation of XRD intensities

A 2D geometric model composed of circles and rectangles is built for the simulation of the SAXS results of compound **1** (see Supplementary Figure 13). The size is estimated using the volume fraction and the electron density is calculated using the crystal volume increment, and the electron density of the aliphatic parts of ~ 321 electrons/nm^3 is taken as the background to which the enhancement of the electron density in the circles and rectangles was added. The glycerol groups form the circles with r = 0.65 nm and the highest electron density being ~129 electrons/nm^3. The aromatic groups are divided into 3 segments, i.e. a central rectangle with a = 0.57 nm, b = 0.45 nm and electron density ~157 electrons/nm^3, and two peripheral rectangles with a = 1.14 nm, b = 0.45 nm and electron density ~69 electrons/nm^3.

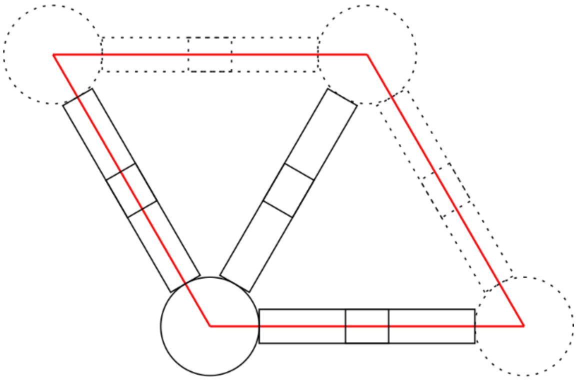

Supplementary Figure 13. Geometric model of hexagonal lattice with triangular tiling pattern.

For a circle with radius a at (0,0), the Fourier Transform (FT) is:

$$\Pi\left(\frac{r}{2a}\right) \overset{\mathrm{FT}}{\Rightarrow} \frac{aJ_1(2\pi aq)}{q}$$ (Supplementary Equation 1)

and for a rectangle centered at (0,0) with length a and width b:

$$\Pi_{ab}(x, y) \overset{\mathrm{FT}}{\Rightarrow} a\mathrm{sinc}(au) \cdot b\mathrm{sinc}(bv)$$ (Supplementary Equation 2)

The FT is a linear transform with:

$$\mathcal{F}(\alpha f + \beta g)\left(\underline{\xi}\right) = \alpha\mathcal{F}f\left(\underline{\xi}\right) + \beta\mathcal{F}g(\underline{\xi})$$ (Supplementary Equation 3)

And the shift and stretch in two-dimensional circumstance is:

$$\mathcal{F}\left(f\left(A\underline{x} - \underline{b}\right)\right) = \exp(-2\pi i\underline{b} \cdot A^{-T}\underline{\xi})\,\frac{1}{|\det A|}\mathcal{F}f(A^{-T}\underline{\xi})$$ (Supplementary Equation 4)

where:

Π – rectangular function

r and q – radius of a radial function in real and reciprocal functions

(x, y) and (u, v) – variables in two-dimensional real and reciprocal functions

f and g – functions

$\mathcal{F}$ – symbol of FT

$\underline{x}$ and $\underline{\xi}$ – spatial variables in real and reciprocal functions

A – rotate matrix, and det A = 1 and $A^{-T} = (A^{-1})^{\mathrm{T}} = (A^{T})^{T} = A$

$\underline{b}$ – shift vector

i – $i = \sqrt{-1}$

Consequently, the relative intensities (which are proportional to $|F|^2$ and normalized to the strongest peak) and the corresponding phases are calculated and listed in Supplementary Table 5.

Supplementary Table 5. Diffraction indices, experimental and simulated intensities, and phases of $\mathrm{Col_{hex\Delta}}$/*p*6*mm* phase of compound **1** using FT of the geometrical model shown in Supplementary Figure 13.

(*hk*)	I_{obs}	I_{simu}	*phase* (simulated)
(10)	66.8	59.5	0
(11)	-	0.04	π
(20)	100.0	100.0	0
(21)	0.3	2.2	π

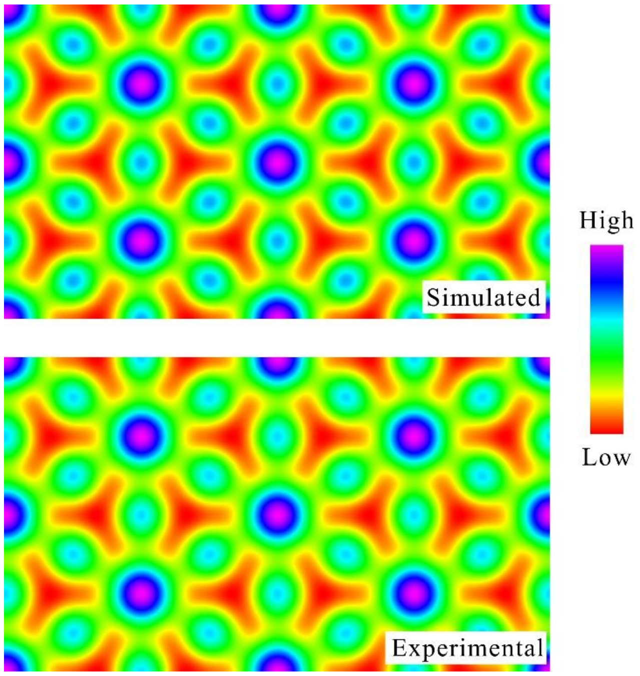

Supplementary Figure 14. Reconstructed electron density maps of $Col_{hex\Delta}$/*p*6*mm* phase of compound **1** using the calculated and experimental intensities, respectveily.

For the hexagonal super-lattice, the same method has been adopted to simulate the intensities. Since the lattice parameter has changed, the size of each segment has been recalculated. The radius of the circle is r = 0.65 nm, and the center rectangle is a = 0.56 nm, b = 0.65 nm and the two peripheral rectangles are a = 1.12 nm, b = 0.65 nm. The geometric model (see Supplementary Figure 15) shows a dodecagonal tiling with *p*6*mm* symmetry.

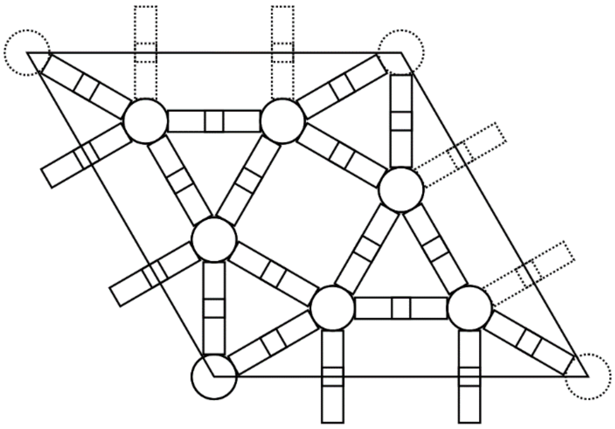

Supplementary Figure 15. Geometric model of hexagonal supper-lattice with triangular and square tiling pattern.

Supplementary Table 6. Diffraction indices, experimental and simulated intensities, and phases of $Col_{hex\Delta/\square}$/*p*6*mm* phase of compound **1** using FT of the geometrical model shown in Supplementary Figure 15.

(*hk*)	I_{obs}	I_{simu}	*phase* (simulated)	*phase* (reconstruction)
(10)	0.2	7.9	0	0
(11)	3.2	6.9	0	0
(20)	41.6	53.5	π	π
(21)	73.6	64.3	0	0
(30)	2.4	0.9	π	π
(22)*	7.9	2.2	0	π
(31)	2.0	0.3	0	0
(40)*	2.8	0.3	0	π
(41)	16.8	34.6	π	π
(50)	42.6	22.3	0	0
(33)	100.0	100.0	0	0
(42)	33.4	30.6	0	0
(51)	6.4	8.3	π	π
(43) *	1.2	17.2	0	π

* The calculated phases are different from the phases using in reconstruction. Since these peaks relatively weak, the main feature mains exactly the same. Supplementary Figure 14 shows the reconstructed electron density map using the calculated phases.

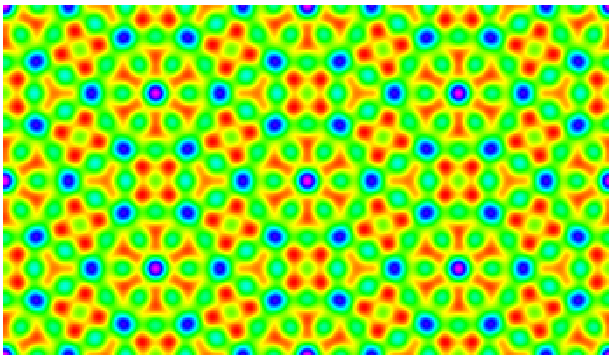

Supplementary Figure 16. Reconstructed electron density map of $Col_{hex\Delta/\square}$/*p*6*mm* phase of compound **1** using the calculated phase combination.

The main difference comes from the geometric model of the super-lattice. The simulations assume a packing of six triangular cells in the hexagonal centers of the dodecagonal supertiles, whereas, as shown in Fig. 5**E** and explained in the main text, the local structure is more likely

composed of alternating rhombs and triangles. Nevertheless, the simulated results strengthen the proposed structure of the hexagonal superlattice.

4.3 Calculation of structural data and development of structural models

Molecular packing parameters. The number of molecules per unit cell with fixed height (0.45 nm for the Col_{hex} phases and 0.60 nm for the $Col_{squ}{}^{T}$ phase) were calculated with two different methods for the distinct models of the phase structures. The first method is based on the crystal volume increments of Immirzi (Supplementary Tables 7-10) and the second one based on the experimentally determined density and molecular mass values (Supplementary Table 11). To determine the density of the substance the floating equilibrium method was used. Compound **1** (10 mg) was melted till no air bubbles appear in the liquid sample. The sample was rapidly cooled down into the glassy state and a globule was formed. A 250 ml beaker was filled with distilled water (100 ml) and the material was put in. Methanol was added with stirring until the material floats in the middle of the liquid mixture (with no stirring). The density of the liquid was determined with a pycnometer. The determined density of the liquid corresponds to the density of **1**, which was thus determines as $\rho = 0.97$ g/cm^3. For the determination of the number of molecules in the unit cells of the hexagonal and square LC-phases the following formula were used:[3]

Hexagonal LC phases

$$n_{cell} = \frac{a_{hex}^2}{2} \cdot \sqrt{3} \cdot h \cdot \frac{N_A}{M} \cdot \rho$$

Square LC phase

$$n_{cell} = a_{squ}^2 \cdot h_{tilt} \cdot \frac{N_A}{M} \cdot \rho$$

N_A = Avogadro constant

M = Molar mass of **1** (1587 g mol^{-1})

Both methods gave almost identical values for the number of molecules per unit cell in the distinct phases (compare Supplementary Tables 10-11).

Supplementary Table 7. Structural data of the $Col_{hex\Delta}/p6mm$ phases.[a]

Comp.	T (°C)	a (nm)	h (nm)	V_{cell} (nm³)	V_{mol} (nm³)	$n_{cell,cr}$	n_{cell}	n_{wall} ∆	n_{wall} ◊
**1**	105	4.15	0.46	6.86	2.45	2.80	2.50	0.83	1.25
**1F**	100	4.16	0.45	6.74	2.50	2.69	2.40	0.80	1.20
**2**	100	4.17	0.46	6.93	2.45	2.83	2.53	0.84	1.26

[a] V_{cell} = volume of the unit cell; for hexagonal phases it is determined according to $V_{cell} = a_{hex}^2/2 \cdot 3^{1/2} \cdot h$, where h is the height of the unit cell, corresponding to the position of the maximum of the diffuse wide angle scattering; V_{mol} = volume of a single molecule as calculated using crystal volume increments;[4] $n_{cell,cr}$ = number of molecules in the unit cell, calculated according to $n_{cell} = V_{cell}/V_{mol}$[5]); n_{cell} = number of molecules per unit cell in the LC state, estimated as 0.893 $n_{cell,cr}$; n_{wall} = number of molecules per wall of the honeycomb structure which is determined according to $n_{wall} = n_{cell}/3$ for the $Col_{hex\Delta}$ phase formed only by triangular cells (∆) and $n_{wall} = n_{cell}/2$ for the Col_{hex} phase formed by rhombic cells (◊).

Supplementary Table 8. Structural data of the giant hexagonal columnar phases $Col_{hex\Delta/\square}/p6mm$.[a]

Comp.	T (°C)	a (nm)	h (nm)	V_{cell} (nm³)	V_{mol} (nm³)	$n_{cell,cr}$	n_{cell}	n_{wall} ∆	n_{wall} ∆/◊	n_{wall} ◊
**1**	75	11.36	0.45	50.29	2.45	20.53	18.33	1.02	1.15	1.22
**1F**	80	11.54	0.45	51.90	2.50	20.76	18.53	1.03	1.16	1.24

[a] For explanations see Table S5. n_{wall} depends on the structure of the hexagon in the centre of the dodecagonal supertiles. It can be composed of: 6 triangles (∆) with $n_{wall} = n_{cell}/18$; 2 triangles and 2 rhombs (∆/◊) with $n_{wall} = n_{cellLC}/16$ or by 3 rhombs (◊) with $n_{wall} = n_{cell}/15$ (see Supplementary Figure 10).

Supplementary Table 9. Structural data of the Col_{squ}^T phase of **1**.[a]

Comp.	T (°C)	a (nm)	β (°)	h_{tilt} (nm)	V_{cell} (nm³)	V_{mol} (nm³)	$n_{cell,cr}$	n_{cell}	n_{wall}
**1**	65	3.29	41.3	0.60	6.49	2.45	2.65	2.37	1.18

[a] For explanations see Supplementary Table 7. For the tilted square phases V_{cell} was determined according to $V_{cell} = a_{squ}^2 \cdot h_{tilt}$, where h_{tilt} is the corrected height of the unit cell and can be determined from $h_{tilt} = h/\cos\beta$. β = tilt angle which can be determined by $\beta = a_{squ}/L_{mol,eff}$, with $L_{mol,eff}$ = 4.38 nm as effective molecular length, previously described.[6]

Supplementary Table 10. Structural data of the different mesophases of compound **1** as determined using Immirzis crystal volume increments.[a]

				$Col_{hex\Delta}$		$Col_{hex\Delta/\square}$			Col_{squ}^T
Phase	a (nm)	h, h_{tilt} (nm)	n_{cell}	n_{wall} ∆	n_{wall} ◊	n_{wall} ∆	n_{wall} ∆/◊	n_{wall} ◊	n_{wall} □
$Col_{hex\Delta}$	4.15	0.46	2.50	0.83	1.25				
$Col_{hex\Delta/\square}$	11.36	0.45	18.33			1.02	1.15	1.22	
Col_{squ}^T	3.29	0.60	2.37						1.18

[a]for explanations, see Supplementary Tables 7-9.

Supplementary Table 11. Structural data of the different mesophases of compound **1** as determined using the measured density and the molecular mass.[a]

				$Col_{hex\Delta}$		$Col_{hex\Delta/\square}$			Col_{squ}^{T}
Phase	*a* (nm)	*h*, h_{tilt} (nm)	n_{cell}	n_{wall} Δ	n_{wall} ◊	n_{wall} Δ	n_{wall} Δ/◊	n_{wall} ◊	n_{wall} □
$Col_{hex\Delta}$	4.15	0.46	2.52	0.84	1.26				
$Col_{hex\Delta/\square}$	11.36	0.45	18.54			1.03	1.16	1.24	
Col_{squ}^{T}	3.29	0.60	2.39						1.20

[a]for explanations, see Supplementary Tables 7-9.

Based on the data in Supplementary Tables 7-11, the number of molecules organized in a section of the cylinder walls with a height of 0.45 and 0.60 nm for the hexagonal and tilted square phases, respectively, (n_{wall}) was calculated assuming different structural models for the $Col_{hex\Delta}$ and $Col_{hex\Delta/\square}$ phases as described in the following.

4.4 Synthesis

4.4.1 General synthesis methods

Diethylmalonate, *n*-bromoctane and 4-benzyloxyphenol were used as obtained from *Sigma-Aldrich*. *n*-Bromodocosane was used as obtained from *abcr*. 4-[4-(4-Ethynylphenylethynyl)phenylmethy]-2,2-dimethyl-1,3-dioxolane (**8**)[7], the branched alkyl bromides **3a and 3b**[8], 4-(4-ethynylphenylethynyl)-3-fluorophenyloxytriisopropylsilane (**14**)[9], 1,4-dihydroxy-2,5-diiodobenzene[10], dimethyl 2-octylmalonate,[11] 2,3-isopropylidene-glycerol-1-*p*-toluenesulfonate (**17**)[12] were synthesized according to the procedure given in the references.

The purity was checked by thin-layer chromatography (TLC, silica gel 60 F254, Merck). Column chromatography was performed with silica gel 60 (0.063-0.2, Merck), flash-chromatography with silica gel 60 (0.040-0.063, Merck). Triethylamine was distilled from CaH_2 and stored over molecular sieve. DMF was stored over molecular sieve.

^{1}H-, ^{13}C-NMR spectra (Varian Unity 500 and Varian Unity 400 spectrometers) were recorded in $CDCl_3$ or pyridine-d_5 solutions, with tetramethylsilane as internal standard). All measurements were operated at 27 ℃.

Elemental analysis was performed using a Leco CHNS-932 elemental analyzer. Mass spectra were recorded with a Bruker HR-ESI-TOF. The measurements were performed in THF (1mg/mL) with 0.1 mg/mL LiCl.

P1: Etherification[13]: A mixture of 1,4-dihydroxy-2,5-diiodobenzene (1 equ.), the appropriate bromoalkane **3a**, **3b** or **12** (2.5 equ.), K_2CO_3 (5 equ.) and Bu_4NI (tip of a spatula) in anhydrous DMF (5 mL per mmol) was stirred at 120 ℃ for 12 h. After cooling to room temperature, the reaction was poured into water (50 mL) and the aqueous layer was extracted with Et_2O (3x50 mL). The combined organic layers were washed with saturated aqu. LiCl, water and brine. After drying over anhydrous Na_2SO_4, filtration and evaporation of the solvent, the crude product was purified by column chromatography.

P2: Sonogashira cross coupling reaction[14]: A mixture of 1,4-dialkoxy-2,5-diiodobenzene (1 equ.) and the appropriate acetylene **8** or **14** (2.1 equ.) was dissolved in dry NEt_3 (5 mL per mmol). After degassing with argon for 30 min [Pd(PPh_3)$_4$] (3 mol%) and CuI (2 mol%) were added and the mixture was refluxed for 6 h. After removing the solvent the obtained residue was purified by column chromatography.

P3: Deprotection of the isopropylidene group with PPTS[15]**:** A mixture of the appropriate isopropylidene acetal **1A**, **2A** or **1FA** (1 equ.) and PPTS (tip of a spatula) was dissolved in THF/MeOH (1:1, 10 mL per 0.1 mmol) and stirred at 50 ℃ for 12 h. After finishing the reaction, the solvent was removed and the residue solved in DCM. The organic layer was washed with $NaHCO_3$ solution (3 x 50 mL), water and brine. After drying over Na_2SO_4 the solvent was removed and the residue purified with column chromatography.

4.4.2 Synthesis of compound 1

1-Benzyloxy-4-(2-octyldec-1-yloxy)benzene (4): Synthesized according to P1 from 4-benzyloxyphenol (3.23 g, 17.4 mmol), **3a** (4.42 g, 13.3 mmol), K_2CO_3 (5.00 g, 39.9 mmol) and Bu_4NI (tip of a spatula) in anhydrous DMF (100 mL). Purification by column chromatography (eluent: $CHCl_3$/*n*-hexane = 1:1). Colourless liquid, $C_{31}H_{48}O_2$, M = 452.37 g/mol, yield: 3.29 g (44%), **^{1}H-NMR** (400 MHz, cdcl$_3$) δ 7.45 – 7.28 (m, 5H, Ar–*H*), 6.92 – 6.87 (m, 2H, Ar–*H*), 6.85 – 6.80 (m, 2H, Ar–*H*), 5.01 (s, 2H, –C*H*$_2$–), 3.77 (d, ^3J(H,H) = 5.7 Hz, 2H, –OC*H*$_2$–), 1.79 – 1.68 (m, 1H, –C*H*–), 1.55 – 1.19 (m, 28H, –C*H*$_2$–), 0.88 (t, ^3J(H,H) = 6.9 Hz, 6H, –C*H*$_3$) ppm.

4-(2-Octyldec-1-yloxy)phenol (5):[16] **4** (3.29 g, 7.5 mmol) was dissolved in 1,4-dioxane (100 mL). Afterwards Pd(OH)$_2$/C (20 w%, catalytic amount) was added and the solution was stirred

at room temperature. The atmosphere was replaced by a hydrogen atmosphere and the solution was stirred for 12 h at 50 °C. After finishing the reaction, the catalyst was filtered off and the solvent was removed under educed pressure. The obtained crude product was purified using column chromatography (eluent: $CHCl_3$) to obtain a colourless liquid. $C_{24}H_{42}O_2$, *M* = 362.32 g/mol, yield: 2.38 g (92%), **^{1}H-NMR** (500 MHz, cdcl$_3$) δ 6.82 – 6.74 (m, 4H, Ar–*H*), 4.33 (s, 1H, –O*H*), 3.77 (d, ^3J(H,H) = 5.7 Hz, 2H, –OC*H*$_2$–), 1.81 – 1.70 (m, 1H, –C*H*–), 1.49 – 1.21 (m, 28H, –C*H*$_2$–), 0.89 (t, ^3J(H,H) = 6.9 Hz, 6H, –C*H*$_3$) ppm.

1-(2-Octyldec-1-yloxy)-4-(2-docosyltetracos-1-yloxy)benzene (6): Synthesized according to P1 from **3b** (0.48 g, 1.4 mmol), **5** (1.00 g, 1.4 mmol), K_2CO_3 (0.95 g, 6.9 mmol) and Bu_4NI (tip of a spatula in DMF (50 mL). Purification by column chromatography (eluent: *n*-hexane). Colourless solid, $C_{70}H_{134}O_2$, *M* = 1007.04 g/mol, mp. 42 °C, yield: 0.65 g (47%), **^{1}H-NMR** (400 MHz, cdcl$_3$) δ 6.81 (s, 4H, Ar–*H*), 3.76 (d, ^3J(H,H) = 5.8 Hz, 4H, –OC*H*$_2$–), 1.81 – 1.72 (m, 2H, –C*H*–), 1.54 – 1.18 (m, 112H, –C*H*$_2$–), 0.88 (t, ^3J(H,H) = 6.8 Hz, 12H, –C*H*$_3$) ppm.

1-(2-Octyldec-1-yloxy)-4-(2-docosyltetracos-1-yloxy)-2,5-diiodobenzene (7):[17] A mixture of **6** (0.65 g, 0.66 mmol), iodine (0.18 g, 0.72 mmol) and $IPh(OCOCF_3)_2$ (0.31 g, 0.72 mmol) in dichloromethane (50 mL) was heated for 12 h under reflux. After finishing the reaction, the solution was cooled to room temperature. The solvent was removed and the obtained crude product was purified by column chromatography (eluent: *n*-hexane) to obtain a colourless solid. $C_{70}H_{132}I_2O_2$, *M* = 1258.83 g/mol, mp. 45 °C, yield: 0.27 g (33%), **^{1}H-NMR** (400 MHz, cdcl$_3$) δ 7.15 (s, 2H, Ar–*H*), 3.80 (d, ^3J(H,H) = 5.4 Hz, 4H, –OC*H*$_2$–), 1.84 – 1.76 (m, 2H, –C*H*–), 1.65 – 1.19 (m, 112H, –C*H*$_2$–), 0.88 (t, ^3J(H,H) = 6.9 Hz, 12H, –C*H*$_3$) ppm.

1-(2-Octyldec-1-yloxy)-4-(2-docosyltetracos-1-yloxy)-2,5-bis{4-[4-(1,2-isopropylidene-1,2-dihydroxyprop-3-yloxy)phenylethynyl]phenylethynyl}benzene (1A): Synthesized according to P2 from **7** (204 mg, 0.17 mmol), **8** (120 mg, 0.36 mmol), $[Pd(PPh_3)_4]$ (6.0 mg, 0.005 mmol) and CuI (0.6 mg, 0.003 mmol) in NEt_3 (50 mL). Purification by column chromatography (eluent: $CHCl_3$). Yellow solid, $C_{114}H_{170}O_8$, *M* = 1667.29 g/mol, mp. 115 °C, yield: 350 mg (96%), **^{1}H-NMR** (500 MHz, $CDCl_3$) δ 7.58 – 7.42 (m, 12H, Ar–*H*), 7.01 (s, 2H, Ar–*H*), 6.94 – 6.88 (m, 4H, Ar–*H*), 4.54 – 4.46 (m, 2H, –OC*H*–), 4.19 (dd, ^3J(H,H) = 8.5 Hz, ^3J(H,H) = 6.4 Hz, 2H, –OC*H*$_2$–), 4.09 (dd, ^3J(H,H) = 9.5 Hz, ^3J(H,H) = 5.4 Hz, 2H, –OC*H*$_2$–), 3.99 (dd, ^3J(H,H) = 9.5 Hz, ^3J(H,H) = 5.9 Hz, 2H, –OC*H*$_2$–), 3.95 – 3.90 (m, 6H, –OC*H*$_2$–),

1.95 – 1.81 (m, 2H, –C*H*–), 1.68 – 1.15 (m, 124H, –C*H*$_2$–, –C*H*$_3$), 0.89 (t, ^3J(H,H) = 6.9 Hz, 6H, –C*H*$_3$), 0.88 (t, ^3J(H,H) = 6.9 Hz, 6H, –C*H*$_3$) ppm.

1-(2-Docosyltetracos-1-yloxy)-4-(2-octyldec-1-yloxy)-2,5-bis{4-[4-(1,2-dihydroxyprop-3-yloxy)phenylethynyl]phenylethynyl}benzene (1): Synthesized according to P3 from **1A** (350 mg, 0.22 mmol) and PPTS (tip of spatula) in MeOH (30 mL) and THF (30 mL). Purification by column chromatography (eluent: $CHCl_3$/MeOH = 9:1) and crystallization from MeOH/THF. Yellow-greenish solid, $C_{108}H_{162}O_8$, *M* = 1587.23 g/mol, yield: 260 mg (75%), **^{1}H-NMR** (500 MHz, pyridine-d$_5$) δ 7.83 – 7.78 (m, 4H, Ar–*H*), 7.76 – 7.70 (m, 4H, Ar–*H*), 7.67 – 7.60 (m, 4H, Ar–*H*), 7.55 (s, 2H, Ar–*H*), 7.15 – 7.06 (m, 4H, Ar–*H*), 4.59 – 4.53 (m, 2H, –OC*H*–), 4.53 – 4.48 (m, 2H, –OC*H*$_2$–), 4.44 – 4.38 (m, 2H, –OC*H*$_2$–), 4.25 – 4.18 (m, 4H, –OC*H*$_2$–), 4.12 (t, ^3J(H,H) = 5.3 Hz, 4H, –OC*H*$_2$–), 2.07 – 1.97 (m, 2H, –C*H*–), 1.82 – 1.70 (m, 4H, –C*H*$_2$–), 1.68 – 1.46 (m, 4H, –C*H*$_2$–), 1.46 – 1.16 (m, 108H, –C*H*$_2$–), 0.88 (t, ^3J(H,H) = 7.0 Hz, 6H, –C*H*$_3$), 0.87 (t, ^3J(H,H) = 7.0 Hz, 6H, –C*H*$_3$) ppm. **^{13}C-NMR** (126 MHz, pyridine-d$_5$) δ 160.07 (–O*C*H$_2$–), 154.36 (–O*C*H$_2$–), 150.00 (–O*C*H$_2$–), 149.21 (–O*C*H$_2$–), 135.66 *C*$_{Ar}$ – H), 135.55, 134.84, 133.38, 133.36, 131.82, 131.79, 131.78, 123.65, 123.55, 122.82, 115.18, 114.31, 95.25 (–C≡C–), 92.46 (–C≡C–), 88.93 (–C≡C–), 88.30 (–C≡C–), 72.40, 71.12, 70.87, 64.04, 38.43, 31.93, 31.91, 31.73, 30.26, 30.24, 29.82, 29.80, 29.79, 29.77, 29.71, 29.45, 29.40, 27.11, 27.07, 22.75, 22.72 (–*C*H$_2$–), 14.08 (–*C*H$_3$), 14.06 (–*C*H$_3$) ppm. HRMS (m/z): [M]+Li$^+$-calcd. for $C_{108}H_{162}O_8Li$, 1594.242; found 1594.245. Anal. Calcd. for $C_{108}H_{162}O_8 \cdot H_2O$: C, 80.75; H, 10.29. Found: C, 80.35; H, 10.04.

4.4.3 Synthesis of compound 2

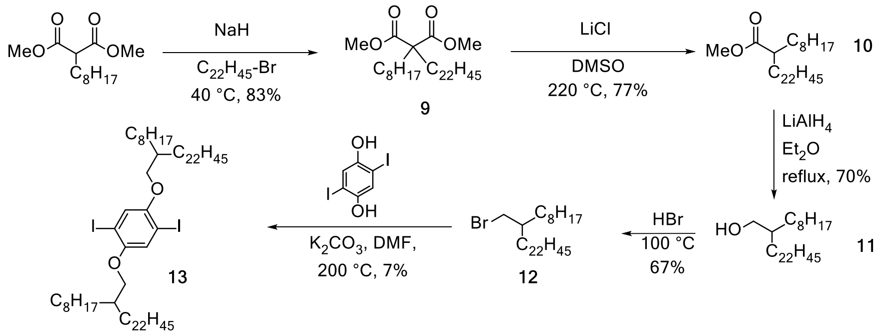

Supplementary Figure 17. Synthesis of the 1,4-dialkoxy-2,5-diiodobenzene **13**.

Dimethyl 2-octyl-2-docosylmalonate (9):[18] The reaction was carried out under an argon atmosphere. Sodium hydride (3.50 g, 88.0 mmol, 60% in mineral oil) was slowly suspended in

DMF (abs., 100 mL) and the mixture was cooled to 0 ℃. Dimethyl 2-octylmalonate (10.00 g, 44.0 mmol) and *n*-bromodocosane (22.20 g, 57.2 mmol) were solved in DMF (50 mL) and added one after another. The mixture was stirred at room temperature for 3 h. After reaction water (250 mL) was added and the mixture was extracted with diethyl ether (3 x 100 mL). The combined organic layers were washed with sat. aqu. LiCl, water and brine. After drying over anhydrous Na_2SO_4 the solvent was removed under reduced pressure. The residue was purified by column chromatography (eluent: $CHCl_3$/*n*-hexane = 1:1) to obtain the product as a colourless solid. $C_{35}H_{68}O_4$, *M* = 552.51 g/mol, mp. 48 ℃, yield: 19.62 g (83%), **^{1}H-NMR** (400 MHz, $CDCl_3$) δ 3.70 (s, 6H, –OCH_3), 1.90 – 1.81 (m, 4H, –CH_2–), 1.32 – 1.09 (m, 52H, –CH_2–), 0.88 (t, ^3J(H,H) = 6.9 Hz, 3H, –CH_3), 0.87 (t, ^3J(H,H) = 6.9 Hz, 3H, –CH_3) ppm.

Methyl 2-octyltetracosanoate (10):[19] A mixture of **9** (19.62 g, 36.6 mmol), LiCl (2.02 g, 47.6 mmol), and water (0.86 g, 47.6 mmol) in DMSO (100 mL) was stirred at reflux for 24 h. After cooling to room temperature water (150 mL) was added. The mixture was extracted with diethyl ether (3 x 50 mL) and the combined organic layers washed with water (3 x 50 mL). After drying over anhydrous Na_2SO_4 the solvent was removed under reduced pressure. The residue was purified by column chromatography (eluent: $CHCl_3$/*n*-hexane = 1:1) to obtain a colourless solid. $C_{33}H_{66}O_2$, *M* = 494.51 g/mol, mp. 43 ℃, yield: 13.07 g (77%), **^{1}H-NMR** (400 MHz, $CDCl_3$) δ 3.66 (s, 3H, –OCH_3), 2.37 – 2.28 (m, 1H, –C*H*–), 1.64 – 1.17 (m, 58H, –CH_2–), 0.88 (t, ^3J(H,H) = 6.8 Hz, 3H, –CH_3), 0.88 (t, ^3J(H,H) = 6.9 Hz, 3H, –CH_3) ppm.

2-Octyltetracosane-1-ol (11):[20] The reaction was carried out under an argon atmosphere. $LiAlH_4$ (1.10 g, 28.8 mmol) was slowly suspended in dry diethyl ether (100 mL). **10** (13.07 g, 24.4 mmol) was dissolved in dry diethyl ether (50 mL) and added dropwise to the suspension. The mixture was heated to reflux for 6 h. After completion of the reaction water was added dropwise with stirring until the excess of $LiAlH_4$ was destroyed. The precipitate was dissolved by adding H_2SO_4 (10%, 50 mL) dropwise. The mixture was extracted with diethyl ether (3 x 50 mL) and the combined organic layers were washed with sat. aqu. $Na_2S_2O_3$, water and brine. After drying over anhydrous Na_2SO_4 the solvent was removed under reduced pressure and the residue was purified by column chromatography (eluent: $CHCl_3$) and a colourless solid was obtained. $C_{32}H_{66}O$, *M* = 466.51 g/mol, mp. 52 ℃, yield: 8.66 g (70%), **^{1}H-NMR** (400 MHz, $CDCl_3$) δ 3.54 (d, ^3J(H,H) = 5.5 Hz, 2H, –CH_2–OH), 1.50 – 1.42 (m, 1H, –C*H*–), 1.38 – 1.19 (m, 57H, –CH_2–, –O*H*), 0.88 (t, ^3J(H,H) = 6.8 Hz, 6H, –CH_3) ppm.

1-Bromo-2-octyltetracosane (12):[21] **11** (8.66 g, 18.6 mmol), Bu_4NHSO_4 (tip of a spatula) and conc. H_2SO_4 (1 mL) was suspended in HBr (48%, 30 mL) and heated to reflux for 24 h. After cooling to room temperature, the mixture was extracted with diethyl ether (3 x 50 mL). The combined organic layers were washed with water and brine and dried over anhydrous Na_2SO_4. After removal of the solvent the residue was purified by column chromatography (eluent: *n*-hexane) to obtain a colourless solid. $C_{32}H_{65}Br$, *M* = 528.43 g/mol, mp. 42 °C, yield: 6.55 g (67%), **^{1}H-NMR** (400 MHz, $CDCl_3$) δ 3.44 (d, ^3J(H,H) = 4.8 Hz, 2H, –C*H*$_2$–Br), 1.64 – 1.56 (m, 1H, –C*H*–), 1.44 – 1.18 (m, 56H, –C*H*$_2$–), 0.88 (t, ^3J(H,H) = 6.9 Hz, 3H, –C*H*$_3$), 0.88 (t, ^3J(H,H) = 6.9 Hz, 3H, –C*H*$_3$) ppm.

1,4-Bis(2-octyltetracos-1-yloxy)-2,5-diiodobenzene (13): Synthesized according to P1 from 1,4-dihydroxy-2,5-diiodobenzene (1.00 g, 2.7 mmol), **12** (3.10 g, 5.8 mmol), K_2CO_3 (1.80 g, 13.5 mmol) and Bu_4NI (tip of a spatula) in DMF (50 mL). Purification by column chromatography (eluent: *n*-hexane). Colourless solid, $C_{70}H_{132}I_2O_2$, *M* = 1258.83 g/mol, mp. 48 °C, yield: 0.23 g (7%), **^{1}H-NMR** (400 MHz, $CDCl_3$) δ 7.15 (s, 2H, Ar–*H*), 3.80 (d, ^3J(H,H) = 5.4 Hz, 4H, –OC*H*$_2$–), 1.87 – 1.70 (m, 2H, –C*H*–), 1.68 – 1.15 (m, 112H, –C*H*$_2$–), 0.88 (t, ^3J(H,H) = 6.8 Hz, 12H, –C*H*$_3$) ppm.

1,4-Bis(2-octyltetracos-1-yloxy)-2,5-bis{4-[4-(1,2-isopropylidene-1,2-dihydroxyprop-3-yl-oxy)phenylethynyl]phenylethynyl}benzene (2A): Synthesized according to P2 from **13** (230 mg, 0.18 mmol), **8** (127 mg, 0.38 mmol), [Pd(PPh_3)$_4$] (6.2 mg, 0.005 mmol) and CuI (0.7 mg, 0.004 mmol) in NEt_3 (50 mL). Purification by column chromatography (eluent: $CHCl_3$). Yellow solid, $C_{114}H_{170}O_8$, *M* = 1667.29 g/mol, mp. 99 °C, yield: 280 mg (94%), **^{1}H-NMR** (400 MHz, $CDCl_3$) δ 7.51 – 7.43 (m, 12H, Ar–*H*), 7.00 (s, 2H, Ar–*H*), 6.93 – 6.87 (m, 4H, Ar–*H*), 4.53 – 4.44 (m, 2H, –OC*H*–), 4.18 (dd, ^3J(H,H) = 8.5 Hz, ^3J(H,H) = 6.5 Hz, 2H, –OC*H*$_2$–), 4.08 (dd, ^3J(H,H) = 9.6 Hz, ^3J(H,H) = 5.4 Hz, 2H, –OC*H*$_2$–), 3.97 (dd, ^3J(H,H) = 9.6 Hz, ^3J(H,H) = 5.9 Hz, 2H, –OC*H*$_2$–), 3.95 – 3.88 (m, 6H, –OC*H*$_2$–), 1.92 – 1.80 (m, 2H, –C*H*–), 1.65 – 1.14 (m, 124H, –C*H*$_2$–,–C*H*$_3$), 0.88 (t, ^3J(H,H) = 6.9 Hz, 6H, –C*H*$_3$), 0.87 (t, ^3J(H,H) = 6.9 Hz, 6H, –C*H*$_3$) ppm.

1,4-Bis(2-octyltetracos-1-yloxy)-2,5-bis{4-[4-(1,2-dihydroxyprop-3-yloxy)phenylethynyl]-phenylethynyl}benzene (2): Synthesized according to P3 from **2A** (280 mg, 0.17 mmol) and PPTS (tip of spatula) in MeOH (30 mL) and THF (30 mL). Purification by column chromatography (eluent: $CHCl_3$/MeOH = 9:1) and recrystallization from MeOH/THF. Yellow

greenish solid, $C_{108}H_{162}O_8$, *M* = 1587.23 g/mol, yield: 190 mg (70%), **^{1}H-NMR** (400 MHz, pyridine-d_5) δ 7.86 – 7.79 (m, 4H, Ar–*H*), 7.77 – 7.71 (m, 4H, Ar–*H*), 7.68 – 7.62 (m, 4H, Ar–*H*), 7.57 (s, 2H, Ar–*H*), 7.15 – 7.08 (m, 4H, Ar–*H*), 4.62 – 4.49 (m, 4H, –OC*H*–, –OCH_2–), 4.43 (dd, ^3J(H,H) = 9.6 Hz, ^3J(H,H) = 6.3 Hz, 2H, –OCH_2–), 4.28 – 4.18 (m, 4H, –OCH_2–), 4.14 (d, ^3J(H,H) = 5.5 Hz, 4H, –OCH_2–), 2.08 – 1.98 (m, 2H, –C*H*–), 1.85 – 1.70 (m, 4H, –CH_2–), 1.70 – 1.18 (m, 108H, –CH_2–), 0.90 (t, ^3J(H,H) = 6.9 Hz, 6H, –CH_3), 0.89 (t, ^3J(H,H) = 6.8 Hz, 6H, –CH_3) ppm. **^{13}C-NMR** (126 MHz, pyridine-d_5) δ 160.06 (–O*C*H2–), 154.35 (–O*C*H2–), 133.37 (C_{Ar}), 131.82, 131.79, 117.06, 115.18, 114.98, 114.31 (C_{Ar}), 95.25 (–C≡C–), 92.46 (–C≡C–), 88.94 (–C≡C–), 88.31 (–C≡C–), 72.39 (–O*C*H2–), 71.12, 70.87, 64.04 (–O*C*H2–), 38.42 (–C*H*–), 31.94 (–CH_2–), 31.91, 31.76, 31.72, 30.26, 29.82, 29.80, 29.77, 29.76, 29.71, 29.46, 29.39, 27.12, 27.07, 22.75, 22.72, 14.09 (–*C*H3), 14.06 (–*C*H3) ppm. HRMS (m/z): [M]+Li^+-calcd. for $C_{108}H_{162}O_8Li$, 1622.196; found 1622.189. Anal. Calcd. for $C_{108}H_{162}O_8 \cdot H_2O$: C, 80.75; H, 10.29. Found: C, 80.77; H, 10.31.

4.4.4 Synthesis of compound 1F

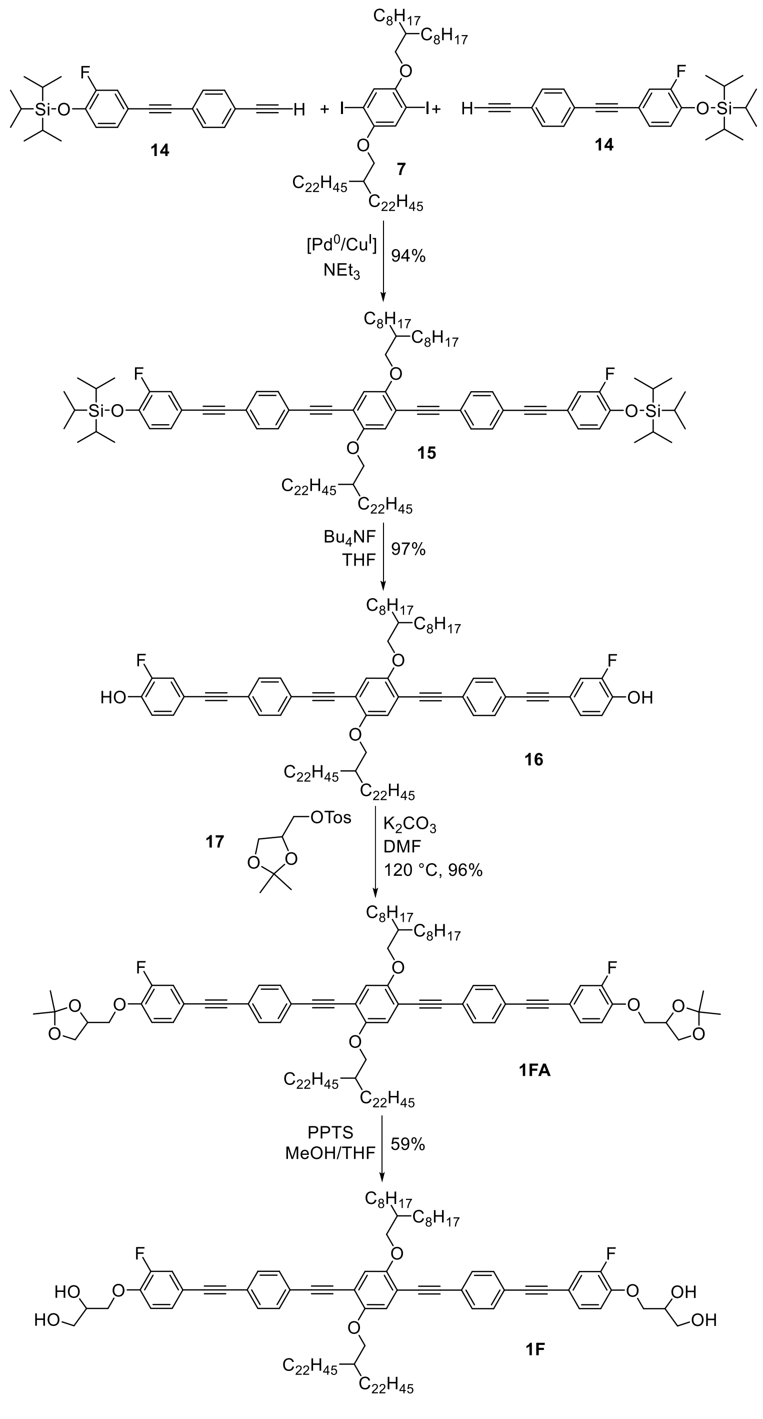

Supplementary Figure 18. Synthesis of compound **1F**.

1-(2-Octyldec-1-yloxy)-4-(docosyltetracos-1-yloxy)-2,5-bis[4-(3-fluoro-4-(triisopropyl-silyloxy)phenylethynyl)phenylethynyl]benzene (15): Synthesized according to P2 from **7** (0.22 g, 0.18 mmol), **14** (0.14 g, 0.37 mmol), [Pd(PPh$_3$)$_2$Cl$_2$] (4.1 mg, 0.005 mmol) and CuI (0.7 mg, 0.004 mmol) in NEt$_3$ (50 mL). Yellow solid, $C_{120}H_{188}F_2O_4Si_2$, *M* = 1787.40 g/mol, mp. 98 °C, yield: 0.30 g (94%), **^{1}H-NMR** (400 MHz, CDCl$_3$) δ 7.57 – 7.43 (m, 8H, Ar–*H*), 7.28 – 7.13 (m, 2H, Ar–*H*), 7.00 (s, 2H, Ar–*H*), 6.91 (pt, ^3J(H,H) = 8.5 Hz, 2H, Ar–*H*), 3.91 (d, ^3J(H,H)

= 6.0 Hz, 4H, –OC*H*$_2$–), 1.91 – 1.79 (m, 2H, –C*H*–), 1.65 – 1.17 (m, 118H, –C*H*–, –C*H*$_2$–,), 1.11 (d, ^3J(H,H) = 7.4 Hz, 36H, –CH(C*H*$_3$)$_2$), 0.88 (t, ^3J(H,H) = 6.8 Hz, 6H, –C*H*$_3$), 0.87 (t, ^3J(H,H) = 6.7 Hz, 6H, –C*H*$_3$) ppm. **^{19}F-NMR** (376 MHz, $CDCl_3$) δ -131.23 – -131.46 (m, Ar–*F*) ppm.

1-(2-Octyldec-1-yloxy)-4-(2-docosyltetracos-1-yloxy)-2,5-bis[4-(3-fluoro-4-hydroxyphenylethynyl)phenylethynyl]benzene (16):[7] **15** (0.30 g, 0.17 mmol) was dissolved in THF (50 mL) and stirred at room temperature. Bu_4NF (0.12 g, 0.39 mmol) was added and stirring continued for additional 1 h. After finishing the reaction, the solvent was removed and to the crude product dichloromethane (20 mL) and water (20 mL) were added. The phases were separated and the aqueous phase was extracted with dichloromethane (3 x 20 mL). The combined organic phases were washed with water and brine. After drying over Na_2SO_4 the solvent was removed and the obtained crude product was purified by column chromatography (eluent: $CHCl_3$) to obtain a yellow solid. $C_{102}H_{148}F_2O_4$, *M* = 1475.13 g/mol, mp. 97 °C, yield: 0.24 g (97%), **^{1}H-NMR** (400 MHz, $CDCl_3$) δ 7.51 – 7.44 (m, 10H, Ar–*H*), 7.38 – 7.32 (m, 2H, Ar–*H*), 7.30 – 7.18 (m, 2H, Ar–*H*), 7.03 – 6.93 (m, 2H, Ar–*H*), 5.32 (d, ^4J(H,H) = 3.3 Hz, 2H, –O*H*), 3.91 (d, ^3J(H,H) = 5.8 Hz, 4H, –OC*H*$_2$–), 1.90 – 1.80 (m, 2H, –C*H*–), 1.61 – 1.17 (m, 112H, –C*H*$_2$–), 0.88 (t, ^3J(H,H) = 6.8 Hz, 6H, –C*H*$_3$), 0.87 (t, $^3J_{H,H}$ = 6.9 Hz, 6H, –C*H*$_3$) ppm. **^{19}F-NMR** (376 MHz, $CDCl_3$) δ -140.38 – -140.52 (m, Ar–*F*) ppm.

1-(2-Octyldec-1-yloxy)-4-(2-docosyltetracos-1-yloxy)-2,5-bis{4-[4-(1,2-isopropylidene-1,2-dihydroxyprop-3-yloxy)-3-fluorophenylethynyl]phenylethynyl}benzene (1FA): Synthesized according to P1 from **16** (240 mg, 0.16 mmol), **17** (110 mg, 0.38 mmol), K_2CO_3 (110 mg, 0.80 mg) and Bu_4NI (tip of a spatula) in DMF (50 mL). Purification by column chromatography (eluent: $CHCl_3$). Yellow solid, $C_{114}H_{168}F_2O_8$, *M* = 1703.27 g/mol, mp. 68 °C, yield: 260 mg (96%), **^{1}H-NMR** (400 MHz, $CDCl_3$) δ 7.59 – 7.43 (m, 8H, Ar–*H*), 7.28 – 7.20 (m, 4H, Ar–*H*), 7.00 (s, 2H, Ar–*H*), 6.96 (pt, ^3J(H,H) = 8.3 Hz, 2H, Ar–*H*), 4.54 – 4.46 (m, 2H, –OC*H*$_2$–), 4.22 – 4.10 (m, 4H, –OC*H*$_2$–), 4.08 – 3.86 (m, 8H, –OC*H*$_2$–), 1.91 – 1.80 (m, 2H, –C*H*–), 1.65 – 1.16 (m, 124H, –C*H*$_2$–, –C*H*$_3$), 0.87 (t, ^3J(H,H) = 6.6 Hz, 6H, –C*H*$_3$), 0.86 (t, (H,H) = 6.9 Hz, 6H, –C*H*$_3$) ppm. **^{19}F-NMR** (376 MHz, $CDCl_3$) δ -133.28 – -133.40 (m, Ar–*F*) ppm.

1-(2-Docosyltetracosan-1-yloxy)-4-(2-octyldec-1-yloxy)-2,5-bis{4-[4-(1,2-dihydroxyprop-3-yloxy)-3-fluorophenylethynyl]phenylethynyl}benzene (1F): Synthesized according to P3 from **1FA** (260 mg, 0.16 mmol) and PPTS (tip of spatula) in MeOH (30 mL) and THF (30 mL).

Purification by column chromatography (eluent: $CHCl_3$/MeOH = 9:1) and recrystallization from MeOH/THF. Yellow greenish solid, $C_{108}H_{160}F_2O_8$, *M* = 1623.21 g/mol, yield: 154 mg (59%), **^{1}H-NMR** (400 MHz, pyridine-d_5) δ 7.87 – 7.81 (m, 4H, Ar–*H*), 7.79 – 7.71 (m, 4H, Ar–*H*), 7.58 (s, 2H, Ar–*H*), 7.54 – 7.44 (m, 2H, Ar–*H*), 7.44 – 7.39 (m, 2H, Ar–*H*), 7.29 – 7.18 (m, 2H, Ar–*H*), 4.64 – 4.55 (m, 4H, –OCH_2–), 4.55 – 4.46 (m, 2H, –OC*H*–), 4.28 – 4.22 (m, 4H, –OCH_2–), 4.14 (d, ^3J(H,H) = 4.5 Hz, 4H, –OCH_2–), 2.10 – 1.97 (m, 2H, –C*H*–), 1.86 – 1.71 (m, 4H, –CH_2–), 1.71 – 1.18 (m, 108H, –CH_2–), 0.89 (t, ^3J(H,H) = 5.9 Hz, 12H, –CH_3) ppm. **^{19}F-NMR** (376 MHz, pyridine-d_5) δ -134.78 – -134.94 (m, Ar–*F*) ppm. **^{13}C-NMR** (126 MHz, pyridine-d_5) δ 152.80 (–O*C*H_2–), 150.52 (d, ^1J(C,F) = 246.2 Hz, C_{Ar}–F), 146.95 (d, ^3J(C,F) = 10.4 Hz, C_{Ar}), 130.32, 130.28, 127.16, 117.64 (d, ^2J(C,F) = 19.7 Hz, C_{Ar}), 115.50 (d, ^5J(C,F) = 1.5 Hz, C_{Ar}), 113.10 (C_{Ar}), 93.62 (–C≡C–), 89.56 (–C≡C–), 87.53 (–C≡C–), 87.24 (–C≡C–), 77.98 (–O*C*H_2–), 70.84, 70.32, 69.44, 62.36 (–O*C*H_2–), 36.87 (–C*H*–), 36.83 (–C*H*–), 30.37 (–CH_2–), 30.35, 30.17, 28.69, 28.68, 28.26, 28.24, 28.23, 28.21, 28.20, 28.15, 27.89, 27.83, 25.55, 25.51, 21.18, 21.16, 12.52, 12.50 (–*C*H_3). HRMS (m/z): [M]+Cl^--calcd. for $C_{108}H_{160}F_2O_8Cl$, 1658.177; found 1658.170.

4.4.4 NMR spectra

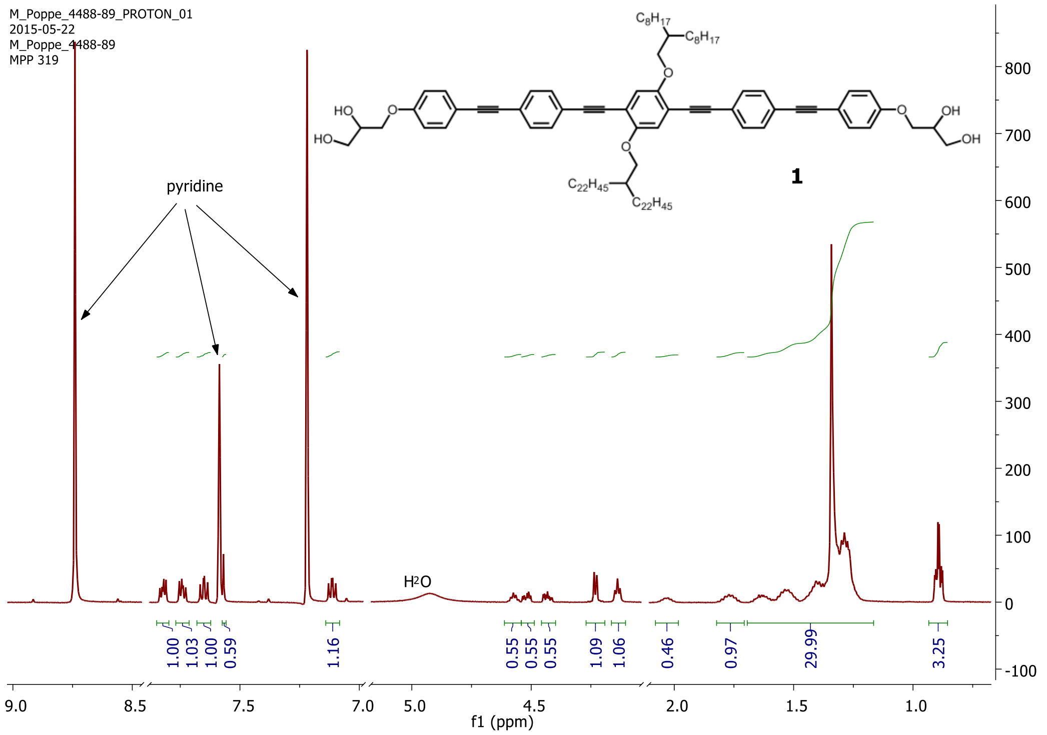

Supplementary Figure 19. ^{1}H-NMR spectra of compound **1** (500 MHz, pyridine-d5).

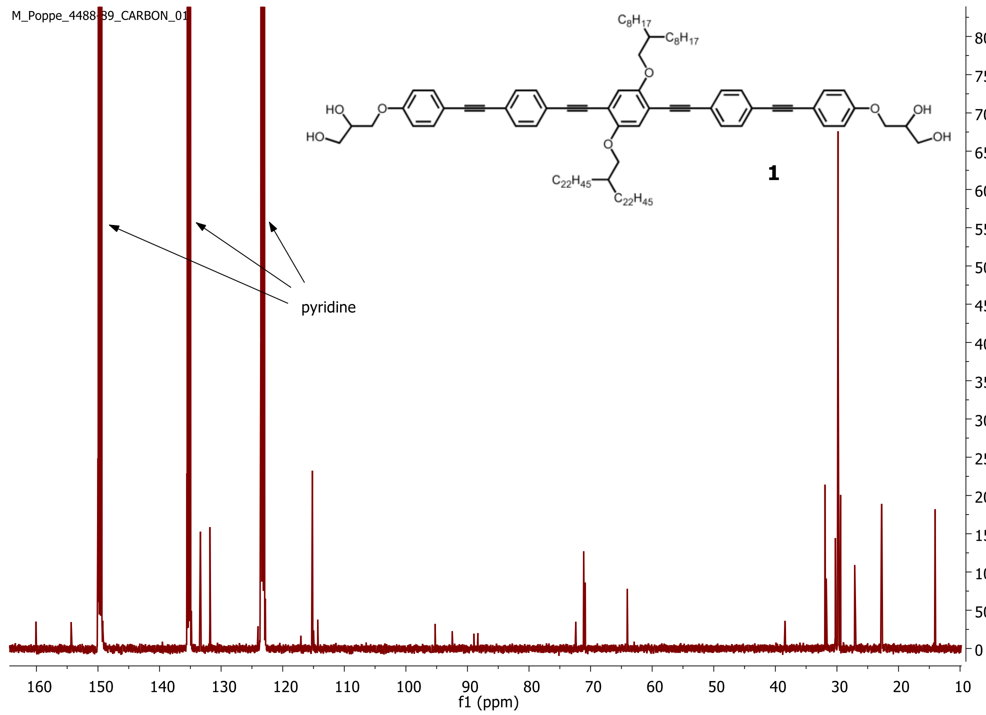

Supplementary Figure 20. ^{13}C-NMR spectra of compound **1** (126 MHz, pyridine-d5).

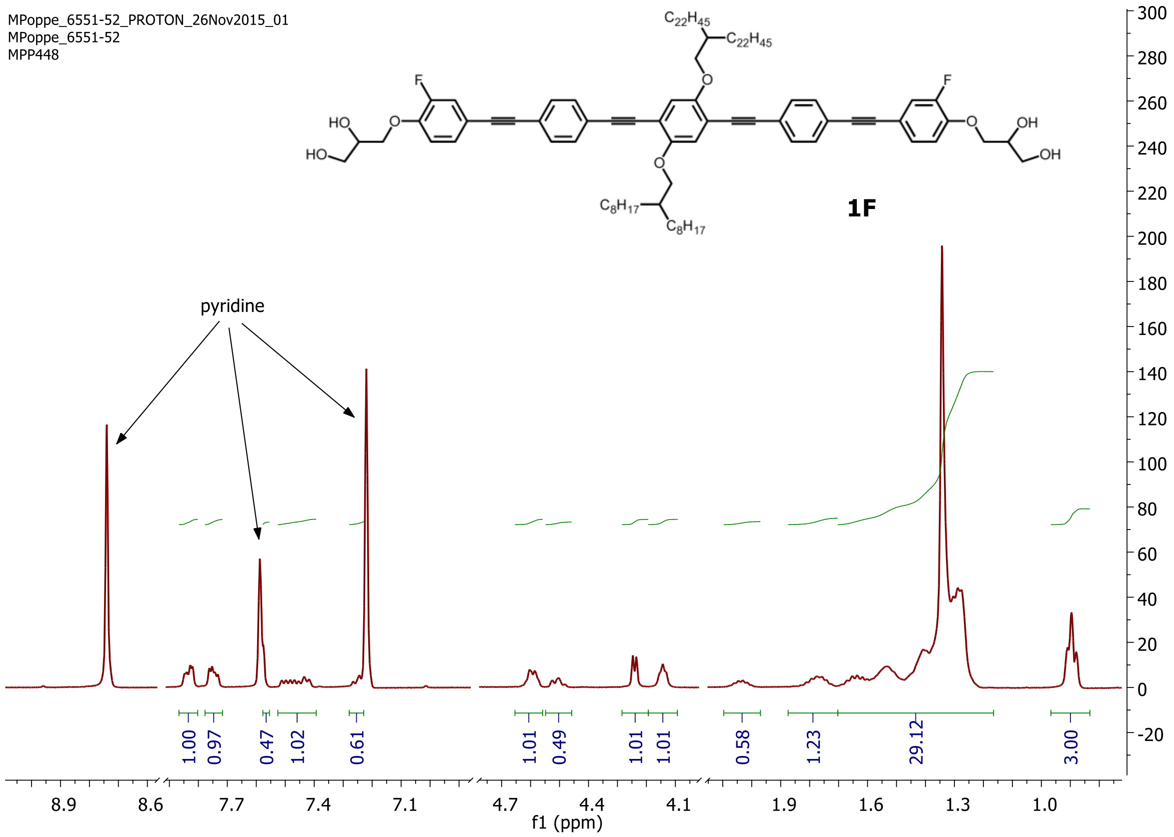

Supplementary Figure 21. ^{1}H-NMR spectra of compound **1F** (400 MHz, pyridine-d$_5$).

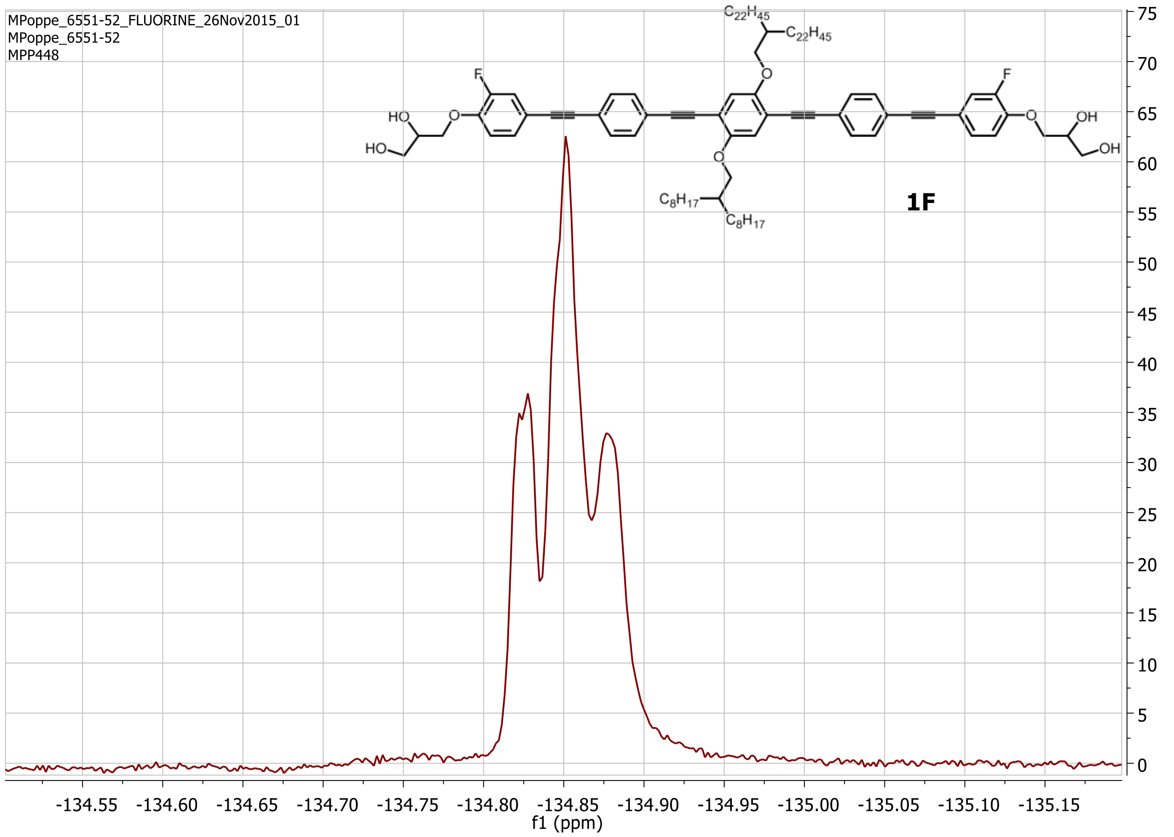

Supplementary Figure 22. ^{19}F-NMR spectra of compound **1F** (376 MHz, pyridine-d$_5$).

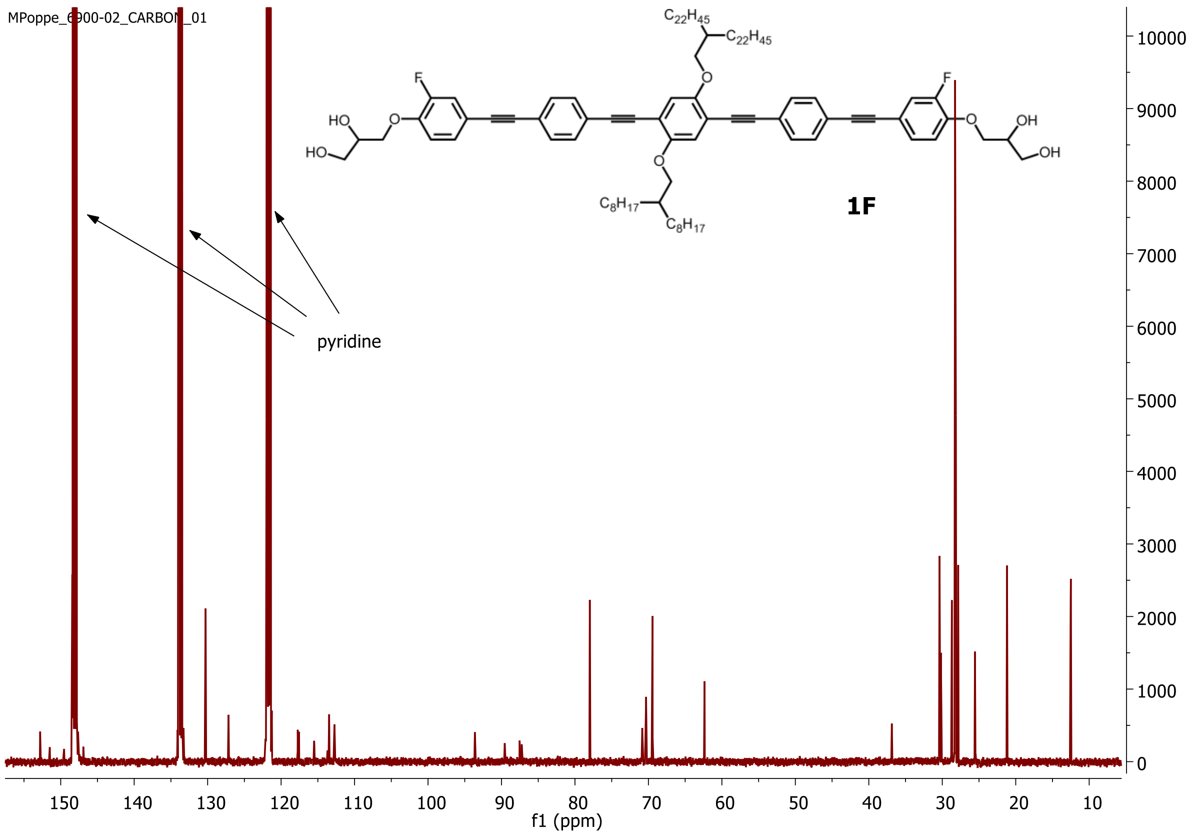

Supplementary Figure 23. ^{13}C-NMR spectra of compound **1F** (126 MHz, pyridine-d$_5$).

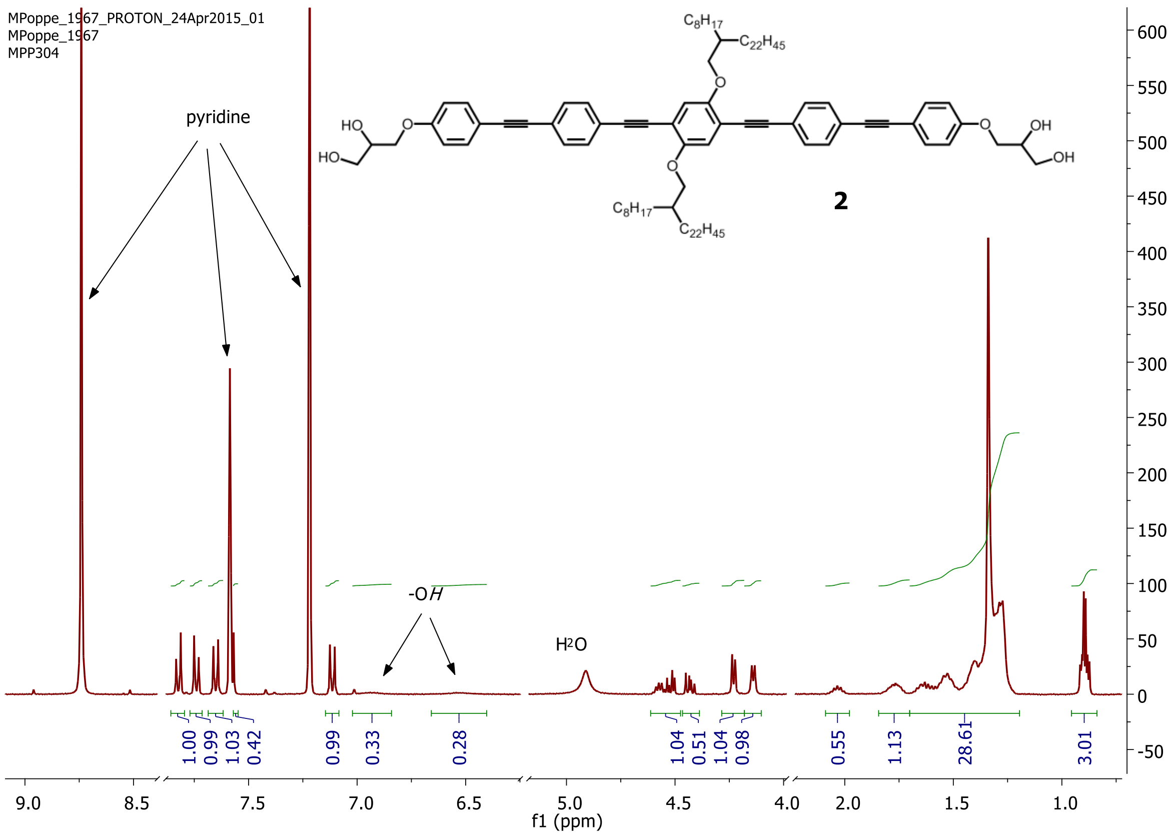

Supplementary Figure 24. ^{1}H-NMR spectra of compound **2** (400 MHz, pyridine-d$_5$).

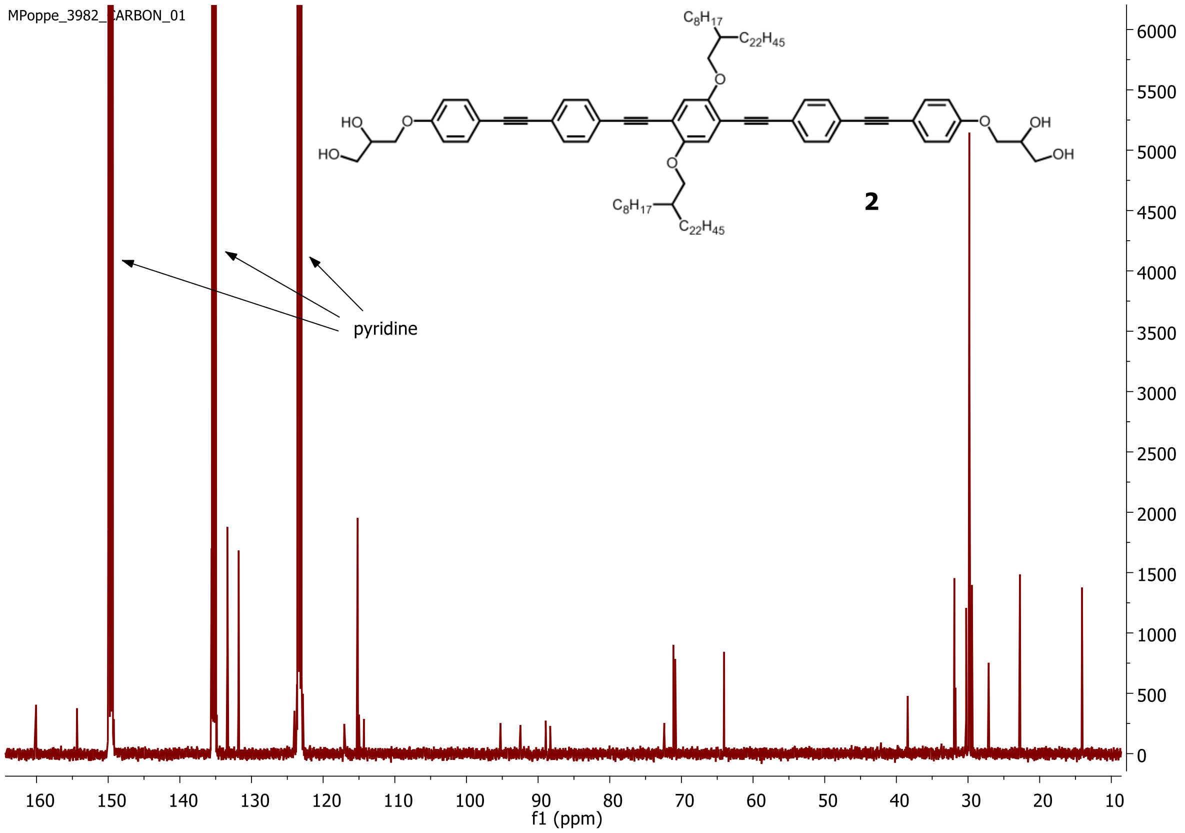

Supplementary Figure 25. ^{13}C-NMR spectra of compound **2** (126 MHz, pyridine-d$_5$).

5. Supplementary References

[1] Werner, S., Ebenhan, J., Poppe, M., Poppe, S., Ebert, H., Tschierske, C. & Bacia, K. Effects of Lateral and Terminal Chains of X-Shaped Bolapolyphiles with Oligo(phenylene ethynylene) Cores on Self-Assembly Behavior. Part 2: Domain Formation by Self-Assembly in Lipid Bilayer Membranes. *Polymers* **2017**, 9, 476.

[2] Cheng, X., Gao, H., Tan, X., Yang, X., Prehm, M., Ebert, H. & Tschierske, C. Transition between triangular and square tiling patterns in liquid-crystalline honeycombs formed by tetrathiophene-based bolaamphiphiles. *Chem. Sci*. **2013**, *4*, 3317–3331.

[3] Borisch, K., Diele, S., Goring, P., Müller, H. & Tschierske, C. Amphiphilic N-benzoyl-1-amino-1-deoxy-D-glucitol derivatives forming thermotropic lamellar, columnar and different types of cubic mesophases. *Liq. Cryst.* **1997**, *22*, 427–443.

[4] Immirzi, A., Perini, B. Prediction of density in organic crystals. *Acta Cryst. Sect. A* **1977**, *33*, 216–218.

[5] Kitaigorodski, A. I. *"Molekülkristalle*", Akademieverlag Berlin, 1979.

[6] Poppe, M., Chen, C., Liu, F., Prehm, M., Poppe, S. & Tschierske, C. Emergence of tilt in square honeycomb liquid crystals. *Soft Matter* **2017**, *13*, 4676-4680.

[7] Werner, S., Ebert, H., Lechner, B.-D., Lange, F., Achilles, A., Bärenwald, R., Poppe, S., Blume, A., Saalwächter, K., Tschierske, C. & Bacia, K. Dendritic Domains with Hexagonal Symmetry Formed by X–Shaped Bolapolyphiles in Lipid Membranes. *Chem. Eur. J.* **2015**, *21*, 8840–8850.

[8] Poppe, S., Poppe, M., Ebert, H., Prehm, M., Chen, C., Liu, F., Werner, S., Bacia, K. & Tschierske, C. Effects of Lateral and Terminal Chains of X-Shaped Bolapolyphiles with Oligo(phenylene ethynylene) Cores on Self-Assembly Behaviour. Part 1: Transition between Amphiphilic and Polyphilic Self-Assembly in the Bulk. *Polymers* **2017**, *9*, 471.

[9] Poppe, M., Chen, C., Liu, F., Poppe, S. & Tschierske, C. Formation of a Cubic Liquid Crystalline Nanostructure with π–Conjugated Fluorinated Rods on the Gyroid Minimal Surface. *Chem. Eur. J.* **2017**, *23*, 7196-7200.

[10] Pawle, R. H., Agarwal, A., Malveira, S., Smith, Z. C. & Thomas, S. W. Bandgap Engineering of Conjugated Materials with Nonconjugated Side Chains. *Macromolecules* **2014**, *47*, 2250–2256.

[11] Kolb, N., Meier & M. A. R. Monomers and their polymers derived from saturated fatty acid methyl esters and dimethyl carbonate. *Green Chem.* **2012**, *14*, 2429-2435.

[12] Fujii, S., Masuno, H., Taoda, Y., Kano, A., Wongmayura, A., Nakabayashi, M., Ito N., Shimizu, M., Kawachi, E., Hirano, T., Endo, Y., Tanatani, A. & Kagechika, H. Boron cluster-based development of potent nonsecosteroidal vitamin D receptor ligands: direct observation of hydrophobic interaction between protein surface and carborane. *J. Am. Chem. Soc.* **2011**, *133*, 20933–20941.

[13] Williamson, A. Theory of ætherification. *Philos. Mag.* **1850**, *3*, 350-356.

[14] Sonogashira, K., Tohda, Y. & Hagihara, N. A Convenient Synthesis of Acetylenes: Catalytic Substitutions of Acetylic Hydrogen with Bromoalkenes, Iodoarenes and Bromopyridines. *Tetrahedron Lett.* **1975**, *50*, 4467–4470.

[15] van Rijsbergen, R., Anteunis, M.-J. & de Bruyn, A. Selective Removal of the Isopropylidene Group in 4-O-Protected 1,6-Anhydro-2,3-O-Isopropylidene-β-D-Mannopyranose and the Conformational Impact of it. *J. Carbohydr. Chem.* **2006**, *2*, 395–404.

[16] Lever Jr., O. W., Bell, L. N., Hymna, C., McGuire, M. & Ferone, R. Inhibitors of dihydropteroate synthase: substituent effects in the side-chain aromatic ring of 6-[[3-(aryloxy)propyl]amino]-5-nitrosoisocytosines and synthesis and inhibitory potency of bridged 5-nitrosoisocytosine-p-aminobenzoic acid analogs. *J. Med. Chem.* **1986**, *29*, 665-670.

[17] Nagarjuna, G., Kokil, A.; Kumar, J. & Venkataraman, D. A straightforward route to electron transporting conjugated polymers. *J. Mater. Chem.* **2012**, *22*, 16091–16094.

[18] Pokholenko, O., Gissot, A., Vialet, B., Bathany, K., Thiéry, A. & Barthélémy, P. Lipidoligonucleotide conjugates as responsive nanomaterials for drug delivery. *J. Mater. Chem. B* **2013**, *1*, 5329-5334.

[19] Krapcho, A. P., Weimaster, J. F., Eldridge, J. M., Jahngen Jr, E. G. E., Loves, A. J. & Stephens, W. P. Synthetic applications and mechanism studies of the decarbalkoxylations of geminal diesters and related systems effected in dimethyl sulfoxide by water and/or by water with added salts. *J. Org. Chem.* **1978**, *43*, 138-147.

[20] Nystrom, R. F. & Brown, W. G. Reduction of Organic Compounds by Lithium Aluminum Hydride. I. Aldehydes, Ketones, Esters, Acid Chlorides and Acid Anhydrides. *J. Am. Chem. Soc.* **1947**, *69*, 1197-1199.

[21] Dakka, G. & Sasson, Y. Selective hydrobromination of branched alcohols using phase transfer catalysis. *Tetrahedron Lett.* **1987**, *28*, 1223-1224.
